# SAR92 clade bacteria are potentially important DMSP degraders and sources of climate-active gases in marine environments

**DOI:** 10.1128/mbio.01467-23

**Published:** 2023-11-10

**Authors:** Xiao-Yan He, Ning-Hua Liu, Ji-Qing Liu, Ming Peng, Zhao-Jie Teng, Tie-Ji Gu, Xiu-Lan Chen, Yin Chen, Peng Wang, Chun-Yang Li, Jonathan D. Todd, Yu-Zhong Zhang, Xi-Ying Zhang

**Affiliations:** 1 State Key Laboratory of Microbial Technology, Shandong University, Qingdao, China; 2 MOE Key Laboratory of Evolution and Marine Biodiversity, Frontiers Science Center for Deep Ocean Multispheres and Earth System, College of Marine Life Sciences, Ocean University of China, Qingdao, China; 3 Laboratory for Marine Biology and Biotechnology, Laoshan Laboratory, Qingdao, China; 4 School of Life Sciences, University of Warwick, Coventry, United Kingdom; 5 School of Biological Sciences, University of East Anglia, Norwich Research Park, Norwich, United Kingdom; 6 State Key Laboratory of Microbial Technology, Marine Biotechnology Research Center, Shandong University, Qingdao, China; 7 Joint Research Center for Marine Microbial Science and Technology, Shandong University and Ocean University of China, Qingdao, China; University of California, Irvine, California, USA

**Keywords:** SAR92 clade, DMSP catabolic pathway, DddD DMSP lyase, DmdA, distribution

## Abstract

Dimethylsulfoniopropionate (DMSP) is one of Earth’s most abundant organosulfur molecules, which can be catabolized by marine bacteria to release climate-active gases through the cleavage and/or demethylation pathways. The marine SAR92 clade is an abundant oligotrophic group of *Gammaproteobacteria* in coastal seawater, but their ability to catabolize DMSP is untested. Three SAR92 clade strains isolated from coastal seawater in this study and the SAR92 representative strain HTCC2207 were all shown to catabolize DMSP as a carbon source. All the SAR92 clade strains exhibited DMSP lyase activity producing dimethylsulfide (DMS) and their genomes encoded a ratified DddD DMSP lyase. In contrast, only HTCC2207 and two isolated strains contained the DMSP demethylase *dmdA* gene and potentially simultaneously demethylated and cleaved DMSP to produce methanethiol (MeSH) and DMS. In SAR92 clade strains with *dddD* and *dmdA*, transcription of these genes was inducible by DMSP substrate. Bioinformatic analysis indicated that SAR92 clade bacteria containing and transcribing DddD and DmdA were widely distributed in global oceans, especially in polar regions. This study highlights the SAR92 clade of oligotrophic bacteria as potentially important catabolizers of DMSP and sources of the climate-active gases MeSH and DMS in marine environments, particularly in polar regions.

**IMPORTANCE:**

Catabolism of dimethylsulfoniopropionate (DMSP) by marine bacteria has important impacts on the global sulfur cycle and climate. However, whether and how members of most oligotrophic bacterial groups participate in DMSP metabolism in marine environments remains largely unknown. In this study, by characterizing culturable strains, we have revealed that bacteria of the SAR92 clade, an abundant oligotrophic group of *Gammaproteobacteria* in coastal seawater, can catabolize DMSP through the DMSP lyase DddD-mediated cleavage pathway and/or the DMSP demethylase DmdA-mediated demethylation pathway to produce climate-active gases dimethylsulfide and methanethiol. Additionally, we found that SAR92 clade bacteria capable of catabolizing DMSP are widely distributed in global oceans. These results indicate that SAR92 clade bacteria are potentially important DMSP degraders and sources of climate-active gases in marine environments that have been overlooked, contributing to a better understanding of the roles and mechanisms of the oligotrophic bacteria in oceanic DMSP degradation.

## INTRODUCTION

The SAR92 clade, belonging to the *Porticoccaceae* family in the *Cellvibrionales* order of the *Gammaproteobacteria* class, is a member of the oligotrophic marine Gammaproteobacteria group (1–3). Although it is also found in pelagic waters, the SAR92 clade is most widely distributed in offshore regions, and its abundance can even reach 10% of total bacteria in nearshore surface seawaters (2). Studies on representative strains, e.g., HTCC2207, indicate that SAR92 clade bacteria are typical marine oligotrophs showing no growth under high nutrient conditions (1). Due to this characteristic, the SAR92 clade bacteria are hard to culture, and few cultivable strains have been reported. The SAR92 clade is found to be one of the most abundant and metabolically active bacterial groups during planktonic algal blooms (4–10), implying that this group has the ability to specifically degrade and utilize organic matter produced and released by algae. Recent studies on the SAR92 clade cultivable strain HTCC2207 have confirmed that it can degrade xylan and laminarin, two polysaccharides prominently produced by phytoplankton (11). However, there is no direct experimental evidence so far that the SAR92 clade is capable of degrading other algae-derived organic matter.

Dimethylsulfoniopropionate (DMSP) is a low molecular weight organosulfur compound produced to millimolar (mM) intracellular levels by many marine phytoplankton, bacteria, corals, and some plants as an antistress compound (12). These organisms produce >10^9^ tons of DMSP annually in Earth’s surface waters, with potentially more being produced in its aphotic waters and sediments (13, 14). Marine DMSP catabolism mainly by bacteria is an important source of reduced carbon and/or sulfur (15, 16) and of the climate-active gases dimethylsulfide (DMS) and methanethiol (MeSH) via DMSP cleavage and demethylation pathways, respectively (17, 18). DMS is the major biogenic sulfur source emitted from the oceans to the atmosphere (19) and with MeSH it is a source of cloud condensation nuclei that influence global sulfur cycling (20), atmospheric chemistry, and potentially climate (21). DMSP and its catabolites are also potent signaling molecules that impact chemotaxis and predator-prey interactions (22–24).

**Fig 1:**
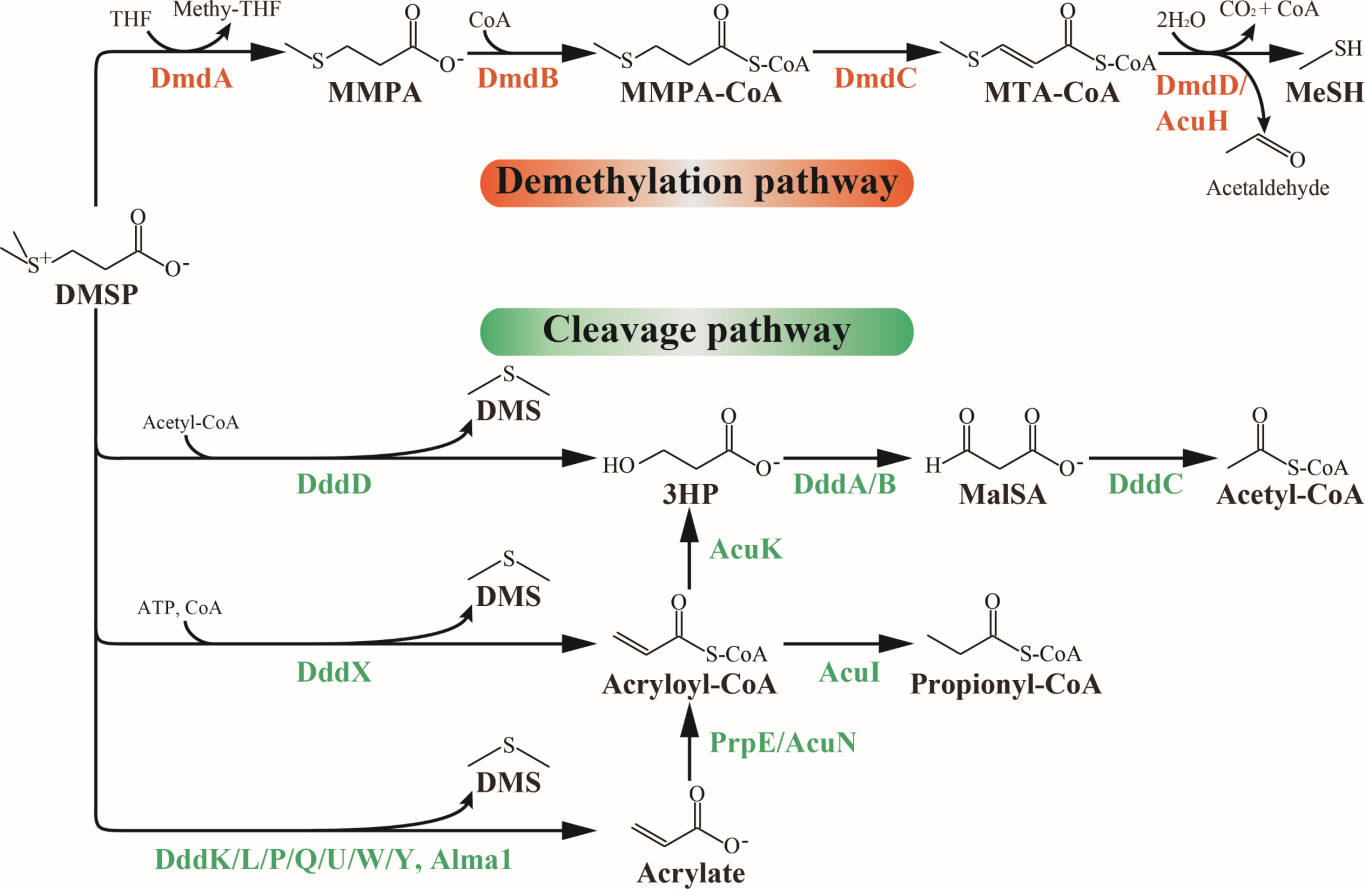
The demethylation and cleavage pathways for DMSP degradation. Enzymes involved in the demethylation and cleavage pathways are shown in orange and green, respectively. In the demethylation pathway, DmdA first demethylates DMSP to generate MMPA with THF as the methyl acceptor. MMPA is successively catabolized by DmdB, DmdC, and DmdD (or AcuH) to generate acetaldehyde and MeSH, forming the intermediates MMPA-CoA and methylthioacryloyl-CoA (MTA-CoA). In the cleavage pathway, DddD cleaves DMSP to produce 3-hydroxypropionate (or 3HP-CoA) and DMS using acetyl-CoA as a cofactor. 3HP is then converted to malonate semi-aldehyde (Mal-SA) and finally to acetyl-CoA by DddA/B and DddC, respectively. DddX cleaves DMSP to generate acryloyl-CoA and DMS using CoA and ATP as co-substrates. Other DMSP lyases (DddL, DddP, DddQ, DddW, DddK, DddU, DddY, and Alma1) cleave DMSP to produce acrylate and DMS. Acrylate is then converted to 3HP by the action of AcuN and AcuK or to propionyl-CoA by the action of PrpE and AcuI.

In the bacterial demethylation pathway (Fig. 1), DMSP is first demethylated to generate 3-methylmercaptopropionic acid (MMPA) by the demethylase DmdA (EC 2.1.1.269), which uses tetrahydrofolate (THF) as the methyl acceptor (25). Then, MMPA is successively catabolized by MMPA-CoA ligase DmdB (EC 6.2.1.x), dehydrogenase DmdC (EC 1.1.1.x), hydrase DmdD (EC 4.2.1.x), or the DmdD ortholog AcuH, to yield acetaldehyde and MeSH for the bacterial utilization (25).

In the cleavage pathway (Fig. 1), DMSP is cleaved by DMSP lyases to generate DMS and acrylic acid, acryloyl-CoA, or 3-hydroxypropionate (3HP) depending on the enzyme. Currently, nine different bacterial DMSP lyases (DddP, DddL, DddQ, DddW, DddK, DddY, DddD, DddX, and DddU) and only algal-specific DMSP lyase (Alma1) have been identified (26–28). These enzymes belong to different protein families with significant structural and biochemical differences (26–28). Many DMSP-degrading *Gammaproteobacteria* possess DddD, a type III acyl-Coenzyme A (CoA) transferase family DMSP lyase that requires acetyl-CoA as a cofactor to generate DMS and 3HP/3HP-CoA from DMSP (29–32). In these bacteria, *dddD* is often in a gene cluster with ancillary genes to enable the import of DMSP from the environment, the metabolism of 3HP into central metabolism, and the regulated expression of the gene cluster (17, 30, 32).

Most known DMSP-degrading bacteria are *Alphaproteobacteria* and *Gammaproteobacteria*, of which the *Roseobacter* and SAR11 clades and the *Oceanospirillales*, respectively, are the most abundant and/or are the major DMSP-catabolizing bacterial groups in marine environments (32, 33). Indeed, many of these diverse DMSP-degrading bacteria, e.g., SAR11 bacteria with *dddK*, can simultaneously demethylate and cleave DMSP (17, 34–37).

Analysis of several gammaproteobacterial SAR92 metagenome-assembled genomes (MAGs) from algal bloom samples revealed that they carry candidate *dddD* and/or *dmdA* genes (38–40), supporting the hypothesis that SAR92 clade bacteria may catabolize DMSP. Given the high abundance and metabolic activity of SAR92 clade bacteria in algal blooms, and their potential to degrade DMSP, it is speculated that these bacteria are globally important marine DMSP degraders and key sources of climate-active gases, but these traits have never been experimentally confirmed. In this study, we isolated three bacterial strains affiliated with the SAR92 clade from Yellow Sea coastal seawater in China and found that together with the reference strain HTCC2207, they all catabolized DMSP. The DMSP catabolic pathways and enzymes that these SAR92 clade bacteria used were elucidated, as was their potential importance in diverse marine environments through examination of *Tara* and polar ocean metagenome and metatranscriptome databases. The data presented here highlight the potential global importance of SAR92 clade bacteria in oceanic DMSP catabolism via both cleavage and demethylation pathways, in sulfur cycling, and in the production of climate active gases.

## RESULTS

### Isolation and phylogenetic characterization of new SAR92 clade strains

**Fig 2:**
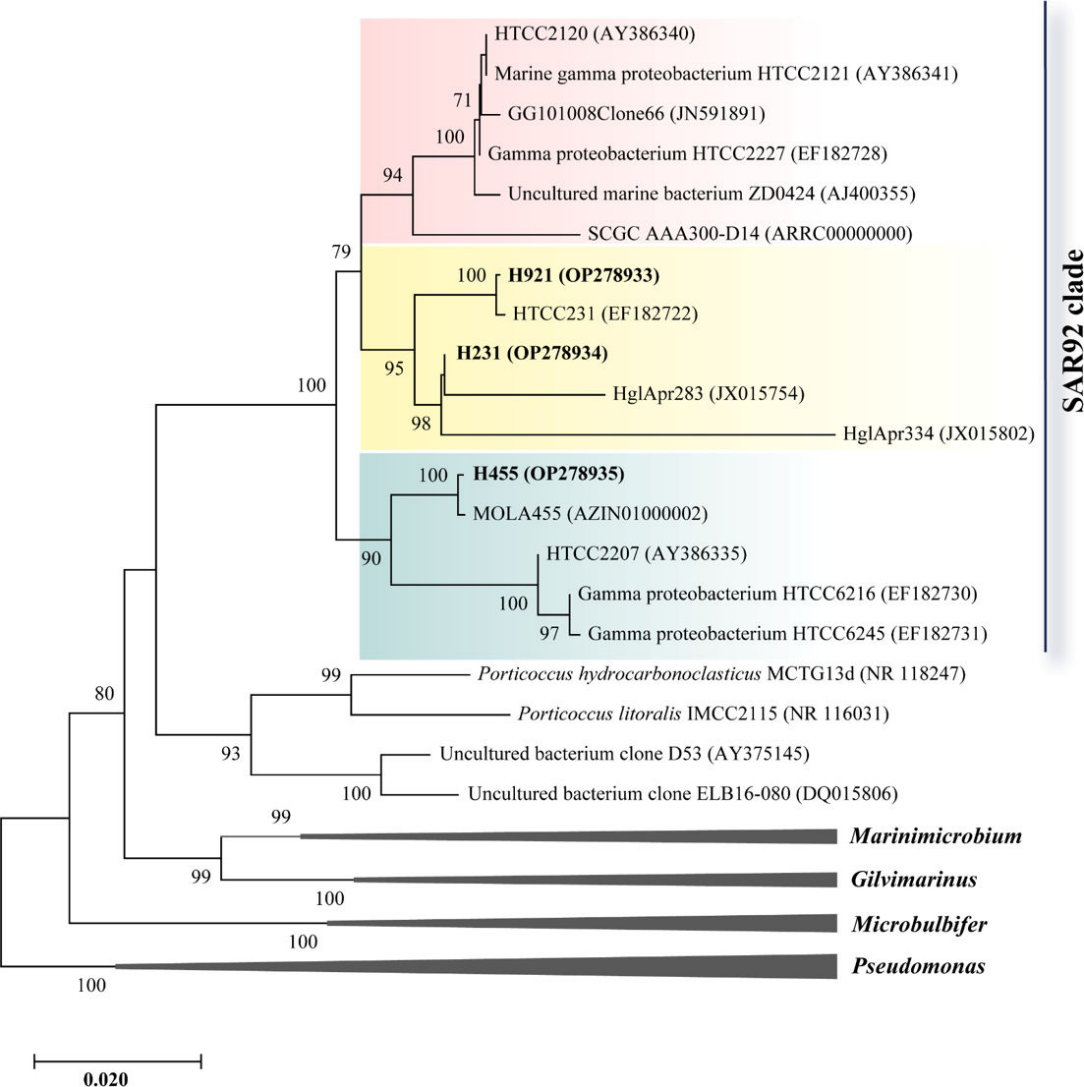
Phylogenetic tree showing the positions of the isolated strains, representative members (or clones) in the SAR92 clade, the family *Porticoccaceae,* and other related genera in the class *Gammaproteobacteria*. The tree was built by the neighbor-joining method with a Kimura 2-parameter model based on the 16S rRNA gene sequences. Bootstrap analysis of 1,000 replicates was conducted, and values above 70% are shown at the nodes. Three subclusters, A (red), B (green), and C (yellow), of the SAR92 clade were shown in different colors in the tree.

Using a high-throughput dilution-to-extinction cultivation method with a low-nutrient medium, 173 bacterial isolates were obtained from Yellow Sea coastal seawater. PCR amplification, sequencing, and phylogenetic analysis of their near complete 16S rRNA genes revealed that most isolates were *Alphaproteobacteria* (46.8%) and *Gammaproteobacteria* (38.7%) (Fig. S1). Three of these isolates belonged to the SAR92 clade (named strains H921, H231, and H455) were selected for further analysis. H921, H231, and H455 shared 97.8%–98.7% 16S rRNA gene sequence identity with each other and 96.4%–97.8% with strain HTCC2207, the SAR92 clade representative strain previously isolated from Oregon coastal seawater (1). Phylogenetic analysis based on the 16S rRNA gene sequences with high bootstrap values (≥90%) support showed that strain H455 was in subcluster B but strains H921 and H231 were in subcluster C of the SAR92 clade (Fig. 2) (2).

### DMSP catabolic phenotypes of the culturable SAR92 clade strains

**Fig 3:**
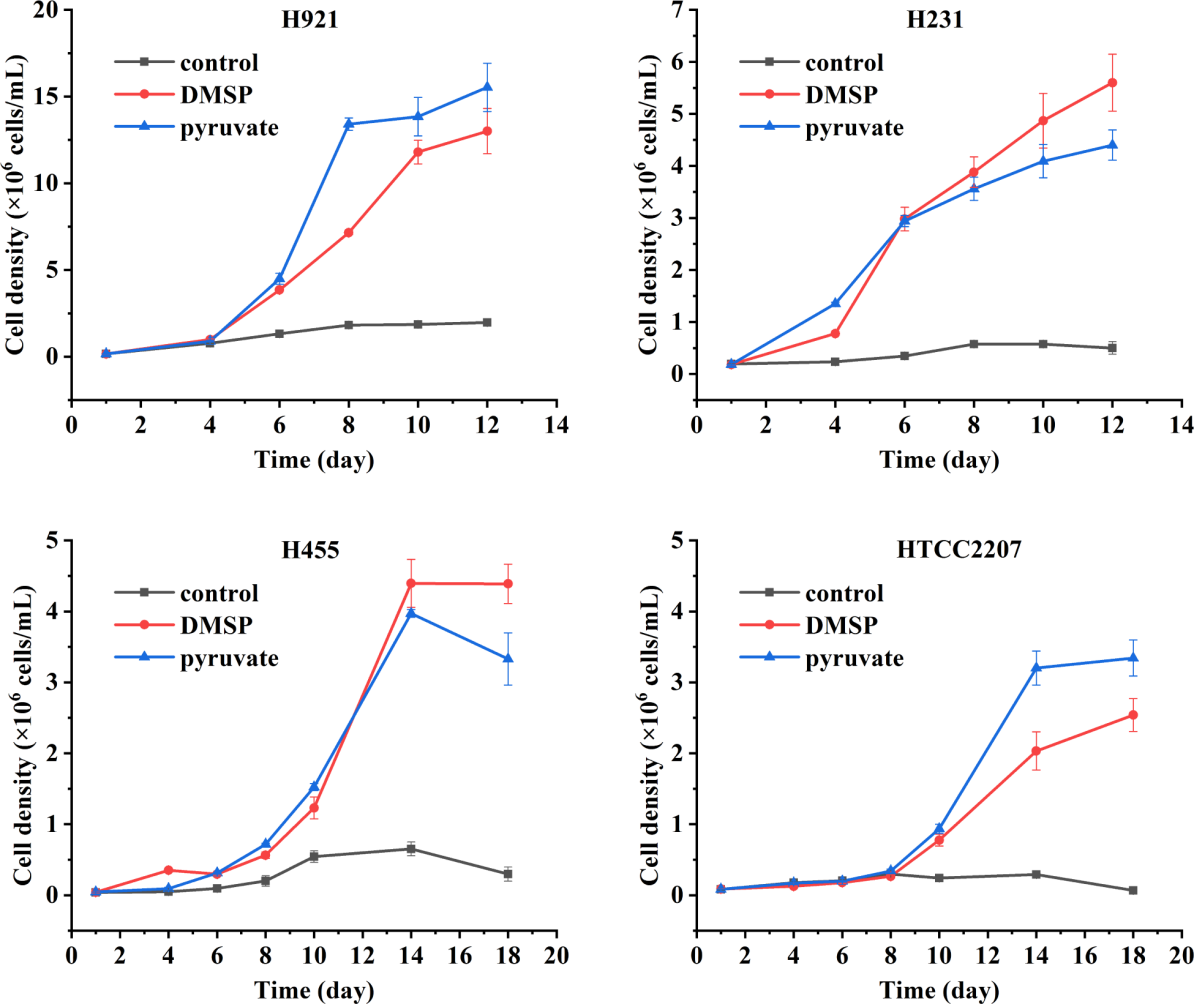
The growth curves of SAR92 strains on DMSP as carbon source. Strains H921, H231, H455, and HTCC2207 were grown at 16°C in the AMS1 medium amended with 50 µM DMSP or pyruvate (positive control) as the sole carbon source. Culture without any carbon source was used as the negative control.

**Fig 4:**
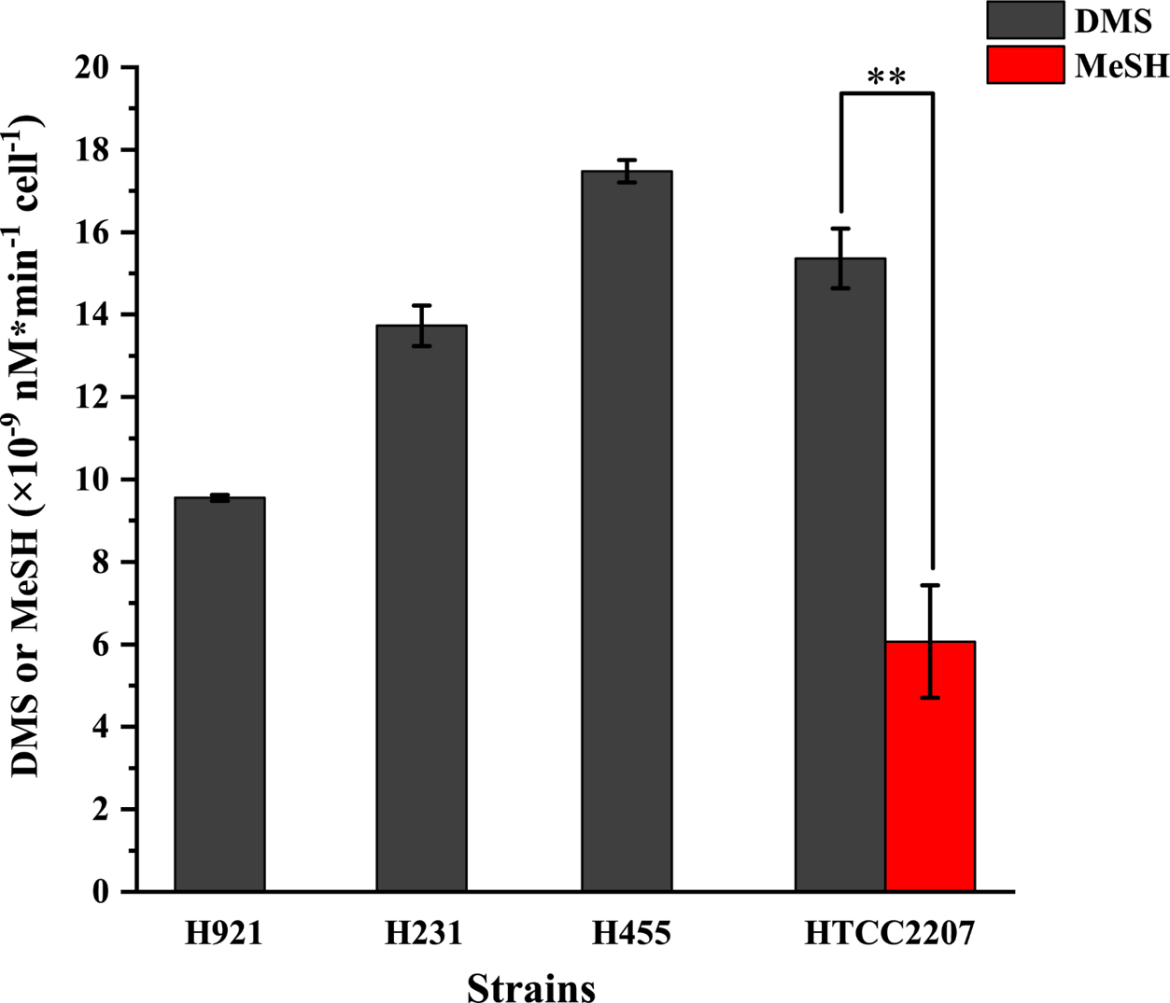
GC detection of DMS and MeSH production from DMSP by culturable SAR92 clade strains. Bacteria were incubated in the AMS1 medium supplied with 1 mM DMSP in a gas-tight sealing bottle at 16°C for 5 days. The culture medium without bacteria was used as the control. A two-sided Student’s *t* test was used to assess statistically significant differences of the products of DMS and MeSH in strain HTCC2207 (**P < 0.01).

The ability of the three isolated SAR92 clade strains, H921, H231, and H455, as well as the representative strain HTCC2207, to catabolize DMSP was then examined. As shown in Fig. 3, all SAR92 clade strains showed noticeable growth phenotypes in a medium containing 50 µM DMSP as the sole carbon source, consistent with them being able to catabolize DMSP. Further analysis of DMSP degradation products showed that all four SAR92 clade strains generated the DMSP cleavage product DMS when incubated with DMSP (Fig. 4). Strain HTCC2207 also produced detectable levels of the demethylation product MeSH when incubated with DMSP, but at significantly less levels (Student’s *t* test, *n* = 3, *P* < 0.01) than that of DMS (Fig. 4). However, strains H921, H231, and H455 produced no detectable levels of MeSH when they were incubated with DMSP. These results imply that all these strains can utilize the DMSP cleavage pathway, whereas strain HTCC2207 may also demethylate DMSP, in which the cleavage pathway likely dominates under the experimental conditions used.

### Genomic analysis of DMSP catabolic pathways in the SAR92 clade strains

To identify DMSP catabolic genes in SAR92 clade strains, the genomes of the strains H921, H231, and H455 were sequenced. The genome sizes of strains H921, H231, and H455 were 2.57, 2.93, and 2.96 Mb in length, respectively, comparable to that of the sequenced strain HTCC2207 (2.63 Mb). The average nucleotide identity between the H921, H231, and H455 genomes ranged between 71.7% and 74.2% and was 72.2% to 84.2% for strain HTCC2207 (Table S1), which were all significantly below the cut-off value (95%–96%) recommended to demarcate bacterial species (41). Thus, strains H921, H231, and H455 and strain HTCC2207 were affiliated with different species in the two subclusters of the SAR92 clade.

**Fig 5:**
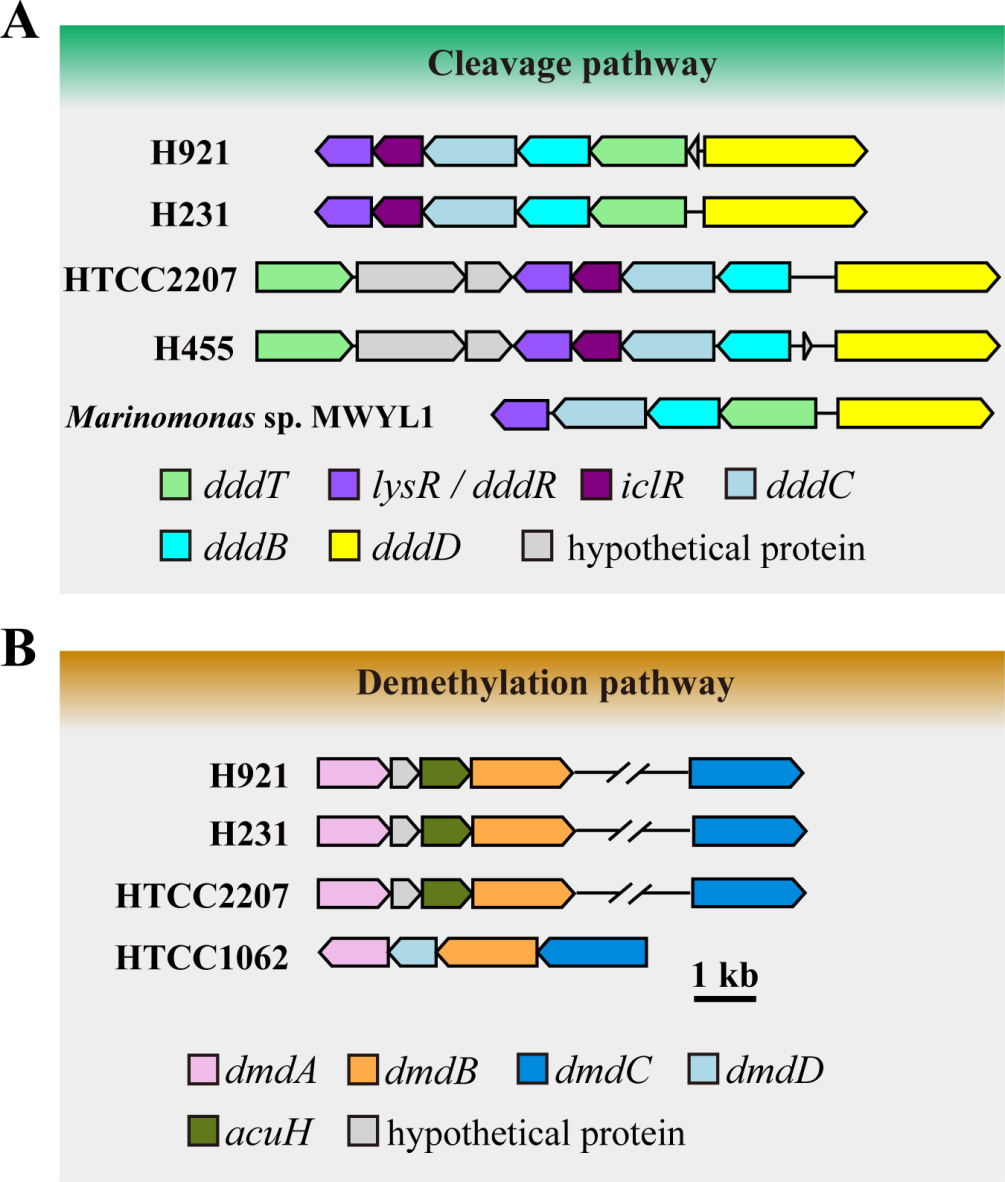
Predicted gene clusters involved in DMSP catabolism from the genomes of the SAR92 clade strains. (**A**) Arrangement of *ddd* genes involved in DMSP catabolism in different strains of the SAR92 clade and *Marinomonas* sp. MWYL1. (**B**) Arrangement of genes involved in DMSP demethylation pathway in different strains of the SAR92 clade and SAR11 clade strain HTCC1062.

Homologous gene searching of strains H921, H231, H455, and HTCC2207 genomes revealed that they all contain a cluster of genes encoding proteins predicted to be involved in the DMSP cleavage pathway (Fig. 5A). Like in other marine *Gammaproteobacteria* that use DMSP as a carbon source, e.g., *Marinomonas* sp. MWYL1 (29, 30, 32), these included a DMSP transporter DddT, candidate LsyR- and IclR-family regulatory proteins, the DMSP lyase DddD, and ancillary proteins DddB and DddD (29, 30) (Table S2; Fig. 5A). The *ddd* gene clusters of strains H921 and H231 had *dddD* divergently transcribed to *dddTBC-iclR-lysR*, similar to those in *Marinomonas* sp. MWYL1 (29). In strains H455 and HTCC2207, *dddD* was divergently transcribed to *dddBC-iclR-lysR,* and *dddT* was directly 3′ of *lysR* but on the opposite DNA strand. No DMSP lyase genes other than *dddD* were predicted from the genomes of these SAR92 clade strains. Consistent with these genetic predictions, all four SAR92 clade strains grew on the DddD co-product 3HP as the sole carbon source (Fig. S2).

Intriguingly, the amino acid sequences of the DddD homologs from the four SAR92 clade strains and many MAGs affiliated with this clade had ~35 amino acid residue long C-terminal extensions and formed taxonomically distinct branches to DddD enzymes from other bacterial groups in phylogenetic analysis (Fig. S3 and S4). These data indicate that the DddD lyases of the SAR92 clade bacteria are distinct in sequence from those of other bacterial groups.

Genes homologous to *dmdA*, *dmdB*, *dmdC*, and *acuH* involved in the DMSP demethylation pathway were also found in the genomes of strains HTCC2207, H921, H231, and H455 (Table S2). These genes were all located in gene clusters with the same gene order (*dmdA-acuH-dmdB-dmdC*) in the genomes of strains HTCC2207, H921, and H231, but were scattered in strain H455 (Fig. 5B). The candidate DmdA proteins from strains HTCC2207, H921, and H231 shared high amino acid sequence identity to each other (73.8%–86.5%) but lower overall identity to the functionally ratified DmdA proteins from *Ruegeria pomeroyi* DSS-3 (37.9%–40.2%) and *Candidatus* Pelagibacter ubique HTCC1062 (34.6%–39.0%) (42, 43). In contrast, the putative DmdA protein from strain H455 shared even lower overall sequence identity with the two ratified DmdAs (21.3%–24.3%) and the candidate DmdA proteins (24.6%–26.4%) from strains HTCC2207, H921, and H231 (Fig. S5). Furthermore, phylogenetic analysis of the ratified DmdA proteins and related proteins from various bacteria showed that the candidate DmdA proteins from strains HTCC2207, H921, and H231 clustered together forming a branch peripheral to known DmdA clades (A–E) (44), whereas the predicted DmdA from strain H455 clustered with a glycine cleavage system protein (GcvT, AAZ21486) from *Candidatus* Pelagibacter ubique HTCC1062, which is distantly related to known DmdA members (Fig. S6). These analyses indicate that the predicted *dmdA* gene in strain H455 may not encode a *bona fide* DmdA enzyme. Consistent with these genetic predictions, strains HTCC2207, H921, and H231 could grow with DMSP as the sole sulfur source, while H455 could not (Fig. S7), confirming that the former three strains contain complete DMSP demethylation pathway, while the latter one lacks it.

Additionally, a hidden Markov model (HMM) search against all 120 MAGs affiliated to the SAR92 clade from different marine samples revealed that 70 MAGs (58%) contain *dmdA*, 66 MAGs (55%) contain *dddD* while 52 MAGs (43%) simultaneously contain *dmdA* and *dddD* (Table S3), suggesting a prevalence of DMSP degradation metabolism in the SAR92 clade bacteria.

### Functional analysis of SAR92 clade DddD and DmdA activity

**Fig 6:**
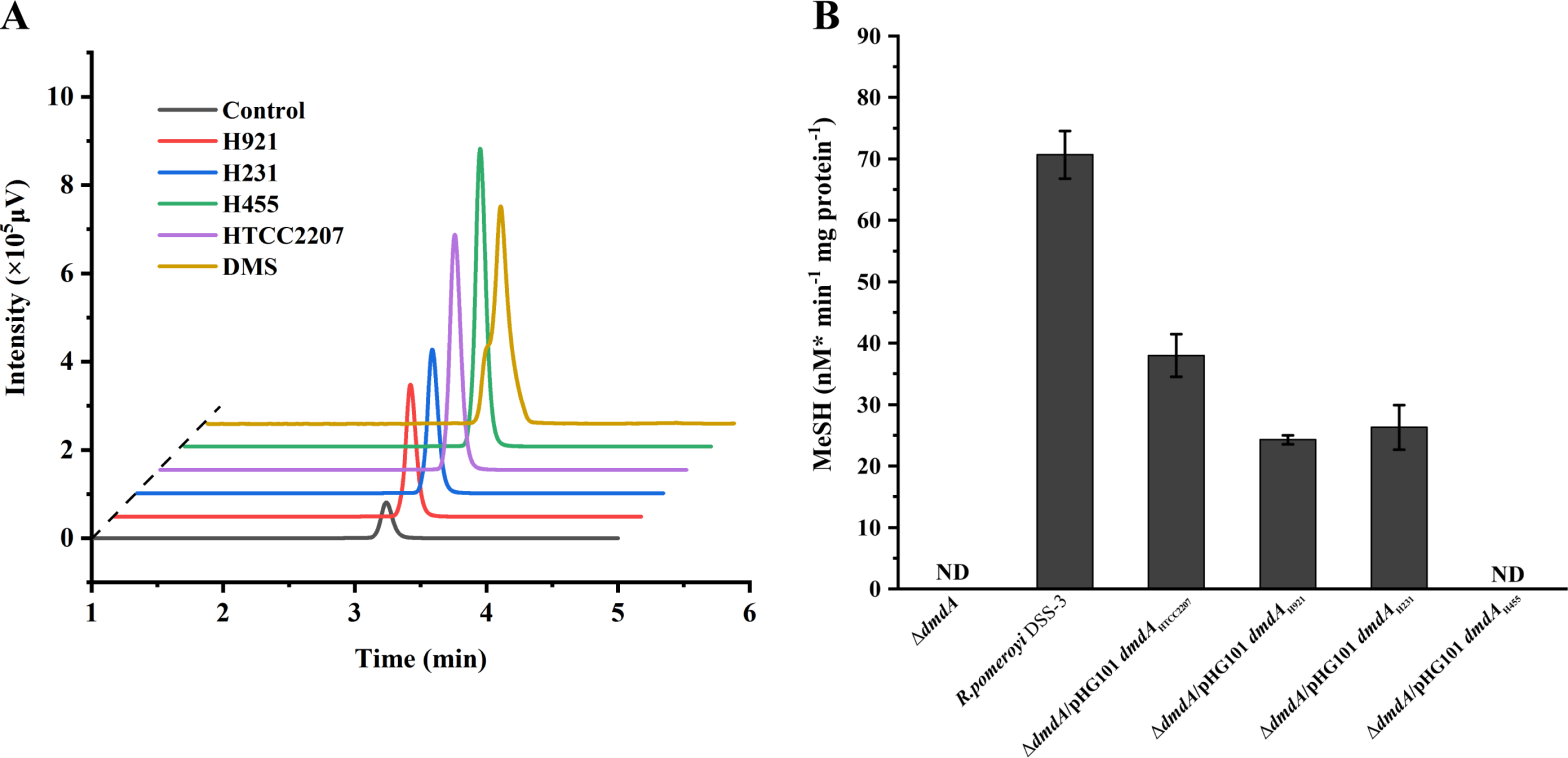
Analyses of the *in vitro* activity of the key enzymes involved in DMSP catabolism in the SAR92 clade strains. (**A**) GC analysis of the enzymatic activity of the recombinant DddD proteins from SAR92 strains. The reaction system without DddD was used as the negative control. The DMS standard was used as the positive control. (**B**) GC analysis of the MeSH production of the *dmdA* complementary strains. The Δ*dmdA* was used as the negative control. The wild-type strain *Ruegeria pomeroyi* DSS-3 was used as the positive control.

To verify the function of the SAR92 clade DddD enzymes in the four cultivable SAR92 clade bacteria, they were expressed and purified as recombinant proteins from *E. coli* BL21(DE3). The recombinant proteins were incubated with DMSP and acetyl-CoA and DMS production was measured by gas chromatography (GC) to report DMSP cleavage activity. As expected, the four recombinant DddD proteins cleaved DMSP to produce DMS (Fig. 6A), showing them as functional DMSP lyase enzymes, which is consistent with the DMSP cleavage phenotypes observed with the four SAR92 clade strains (Fig. 4).

The DMSP demethylation activity of the candidate *dmdA* gene products from the four cultivable SAR92 clade strains was established through genetic complementation of a *Ruegeria pomeroyi* DSS-3 *dmdA*^-^ mutant (Δ*dmdA*), which is unable to generate MeSH from DMSP. When cloned and expressed, the candidate *dmdA* genes from strains HTCC2207, H921, and H231 restored MeSH production in the *R. pomeroyi* DSS-3 *dmdA*^-^ strain at 53.8%, 34.3%, and 37.2% of the wild-type levels, respectively (Fig. 6B). In contrast, the putative *dmdA* from strain H455 did not restore MeSH production (Fig. 6B). Thus, we conclude that the SAR92 clade DmdA proteins from strains HTCC2207, H921, and H231 are functional DmdA proteins but the DmdA-like protein from strain H455 likely has another unknown role.

### Transcriptional analysis of SAR92 clade DMSP catabolic genes

**Fig 7:**
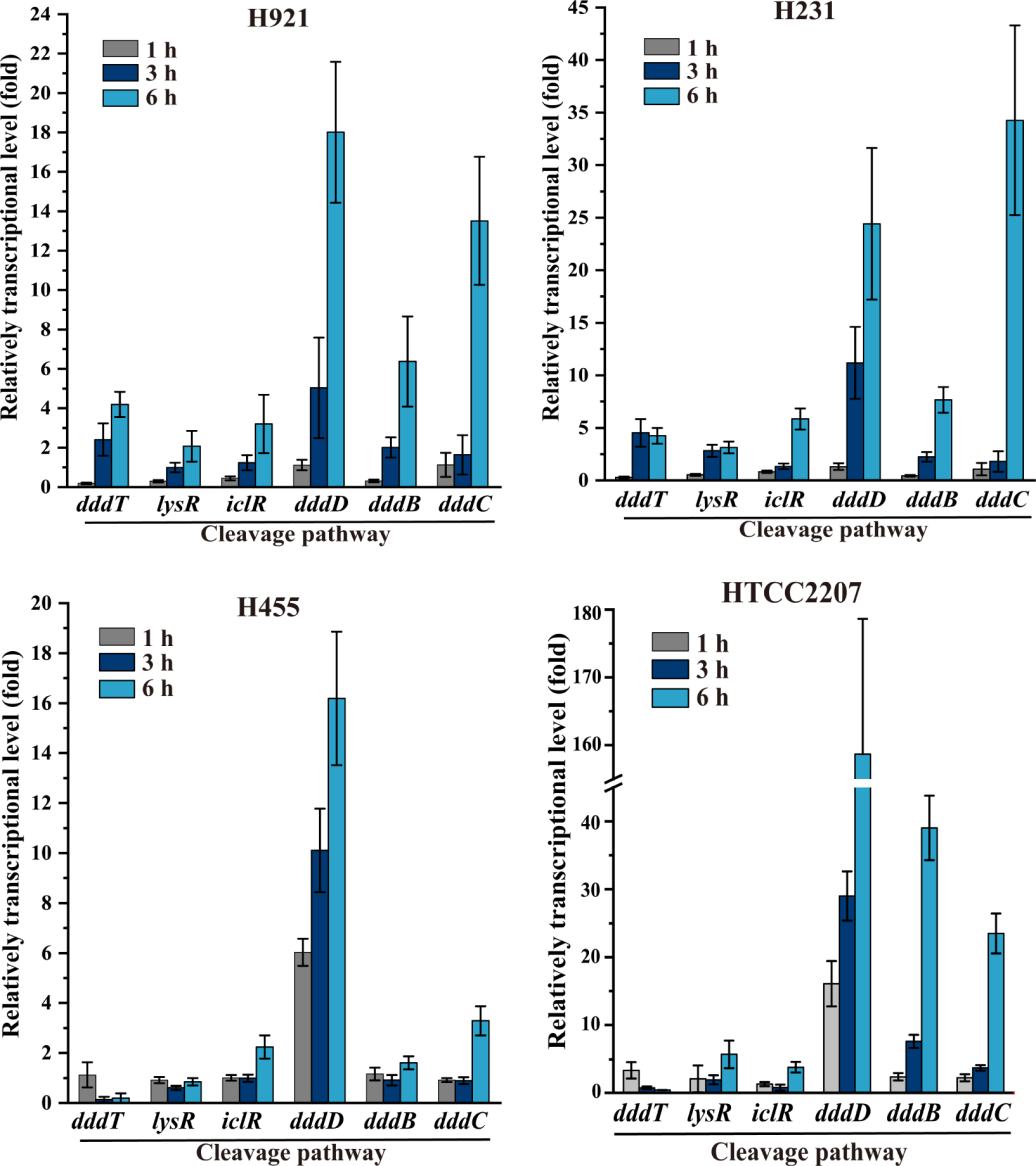
Transcriptions of the genes involved in the DMSP cleavage pathway in the SAR92 clade strains. RT-qPCR assay of the transcriptions of the genes involved in the cleavage pathway from strains H921, H231, H455, and HTCC2207 in response to DMSP. The bacteria cultured in the same medium without DMSP were used as the control. The *recA* gene was used as an internal reference. The error bars represent the standard deviation of triplicate experiments.

In some bacteria, the transcription of *dmdA* and *dddD* was induced by DMSP substrate (29, 45, 46). Real-time qPCR (RT-qPCR) experiments were conducted to examine the transcription of *ddd* (*dddT*, *lysR*, *iclR*, *dddD*, *dddC*, and *dddB*) and demethylation pathway genes (*dmdA*, *dmdB*, *dmdC*, and *acuH*) in the four culturable SAR92 clade strains in response to DMSP. In all SAR92 clade strains, the key *dddD*, *dddC*, and *dddB* genes encoding the structural enzymes of the DMSP cleavage pathway were all upregulated by incubation time with DMSP (by 15–158-fold, 2–33-fold, and 0.6–38-fold after 6 h incubation with DMSP, respectively) (Fig. 1 and 7). Such a coordinated response to DMSP substrate availability is important in bacteria that use DMSP as a carbon source. The regulatory (*lysR*, *iclR*) and transport (*dddT*) genes in the H921 and H231 *ddd* clusters also showed enhanced transcription with DMSP at lower levels (by one- to fourfold after 6 h incubation with DMSP) (Fig. 7). Regulation of *lysR*, *iclR,* and *dddT* transcription by DMSP was not obvious in the H455 and HTCC2207 strains, in which *dddT* is predicted to be transcribed as a single gene.

None of the candidate DMSP demethylation genes (*dmdA*, *dmdB*, *dmdC*, and *acuH*) were upregulated at the transcription level by DMSP in strain H455 (Fig. 1; Fig. S8). These results further support strain H455 as not containing the demethylation pathway and thus not being able to catabolize DMSP via this pathway. In contrast, the transcription of *dmdA*, *dmdB*, *dmdC*, and *acuH* in strain HTCC2207 was significantly upregulated by 2–21-fold after 3 h incubation with DMSP (Fig. S8), consistent with this strain showing DMSP-dependent MeSH production (Fig. 4), being able to grow on DMSP as a carbon and sulfur source (Fig. S7) and thus having an active DMSP demethylation and cleavage pathway. The transcription of *dmdA*s in strains H921 and H231 was also upregulated by one to twofold after 1 h incubation with DMSP, as was that of *dmdB*, *dmdC*, and *acuH* after 3 or 6 h incubation with DMSP (Fig. S8). These data support the notion that strains H921 and H231 have an active DMSP demethylation pathway but that it may not be as effective as that in strain HTCC2207 under our experimental conditions given that these strains liberated no detectable levels of MeSH from DMSP, and the regulation of related genes was less significant than in strain HTCC2207.

### Distribution of the SAR92 clade bacteria and their *dddD* and *dmdA* in the oceans

**Fig 8:**
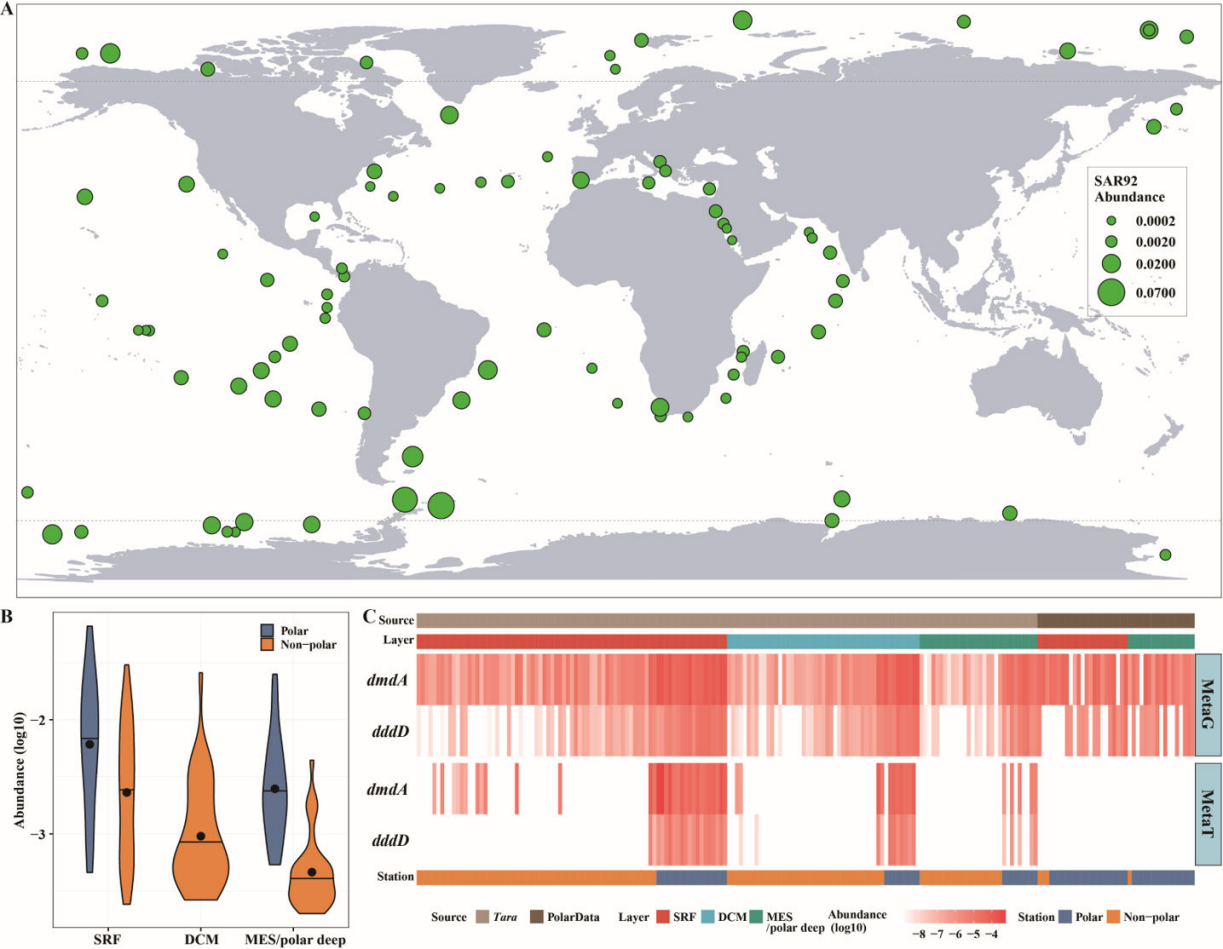
Distribution and abundance of the SAR92 clade bacteria (**A and B**) and their *dddD* and *dmdA* (**C**) in the metagenomes or metatranscriptomes in *Tara* and polar ocean databases. (**A**) Distribution and abundance of the SAR92 clade bacteria in the *Tara* and polar surface seawater metagenomes. (**B**) Abundance of the SAR92 clade bacteria in *Tara* and polar ocean metagenomes across three depth layers. The median is shown as a black horizontal line, and the average value is shown as a black dot in the violin plot. SRF, surface seawater layer; DCM, deep chlorophyll maximum layer; and MES, mesopelagic zone. (**C**) Distribution and abundance of *dddD* and *dmdA* genes belonging to the SAR92 clade in the *Tara* and polar ocean metagenomes (MetaG) or metatranscriptomes (MetaT). PolarData includes metagenomes from the polar ocean project (46). Stations with latitudes greater than or lower than 60^o^ are considered to be in polar or non-polar regions, respectively.

Having experimentally confirmed the capability of the SAR92 clade bacteria to catabolize DMSP through the DddD-mediated DMSP cleavage pathway and, in some cases, through the DmdA-mediated demethylation pathway, we explored the environmental significance of these processes in global oceans by elucidating the abundance, distribution, and transcription of SAR92 clade bacteria and their ratified DMSP catabolic genes in the *Tara* and polar ocean metagenome and metatranscriptome databases. SAR92 clade bacteria were found in all 88 surface seawater metagenomes from global ocean sites at relative abundances of 0.024%–6.58% which, for comparison, was much less than that of the SAR11 clade bacteria from 0.66% to 51.22% (Fig. 8A; Fig. S9 and S10). SAR92 clade bacteria were generally much more abundant (mean value, 1.27%) in polar ocean (|latitude| ≥ 60°) surface seawater metagenomes (26) than in low and middle latitude ocean surface seawater metagenomes (66) (mean value, 0.49%) (Wilcoxon’s rank-sum test, *P* value < 0.001) (Fig. 8B). In addition, SAR92 clade bacteria were significantly more abundant in 88 surface seawater metagenomes than in 104 deep layer seawater ones (Wilcoxon’s rank-sum test, *P* value < 0.001), with an average abundance of 0.72% in the former and 0.25% in the latter. The average abundance (0.30%) of the SAR92 clade bacteria in 39 deep chlorophyll maximum (DCM) layer seawater metagenomes was also much higher than that (0.08%) in 29 mesopelagic zone (MES) seawater ones (Fig. 8B). Overall, these results show SAR92 clade bacteria to be widely distributed and relatively abundant in Earth’s surface oceans.

The distribution and transcription of the SAR92 clade’s *dddD* and *dmdA* in oceans were then investigated using hmmsearch in the *Tara* and polar ocean metagenomes and metatranscriptomes. The *dddD* and *dmdA* genes belonging to the SAR92 clade were detectable in 70 and 99 out of 111 *Tara* and polar ocean surface seawater metagenomes (Fig. 8C; Fig. S11 and Table S4), with an average abundance of 0.00035% and 0.00269%, respectively. Like the distribution of the SAR92 clade bacteria, the abundance of *dddD* and *dmdA* genes belonging to the SAR92 clade in polar ocean surface seawater metagenomes (with an average of 0.00076% in 30 metagenomes for *dddD* and 0.0065% in 35 metagenomes for *dmdA*, respectively) was significantly higher than those in low and middle latitude ocean surface seawater metagenomes (with an average of 0.00004% in 40 metagenomes for *dddD* and 0.00059% in 64 metagenomes for *dmdA*, respectively) (Wilcoxon’s rank-sum test, *P* value < 0.001) (Fig. S12). In addition, the *dddD* and *dmdA* transcripts belonging to the SAR92 clade were also detectable in 42 and 94 out of 102 *Tara* ocean surface seawater metatranscriptomes (Fig. 8C; Fig. S13 and Table S5). They were found to comprise on average 0.00043% and 0.00297%, respectively, of total transcripts in these metatranscriptomes. The *dddD* and *dmdA* transcripts were also much more abundant in polar ocean surface water metatranscriptomes (with an average abundance of 0.00079% in 22 metatranscriptomes for *dddD* transcript and 0.01049% in 22 metatranscriptomes for *dmdA* transcript, respectively) than in low and middle latitude ones (with an average abundance of 0.00004% in 20 metatranscriptomes for *dddD* transcript and 0.00067% in 72 metatranscriptomes for *dmdA* transcript, respectively) (Wilcoxon’s rank-sum test, *P* value < 0.001) (Fig. S12). These results suggest that the SAR92 clade bacteria containing and transcribing DddD and DmdA are also widely distributed in global oceans, especially in polar regions, which, therefore, are likely an important bacterial group involved in global DMSP degradation and cycling.

## DISCUSSION

Many copiotrophic bacteria belonging to diverse genera (e.g., *Marinomonas*, *Halomonas*, *Pseudomonas*, *Altermonas*, and *Vibrio*) of the *Gammaproteobacteria* class have been reported to catabolize DMSP (29, 30, 32, 37). However, it still remains unclear whether and how oligotrophic groups of the *Gammaproteobacteria* class participate in the metabolism of DMSP in marine environments, which is in large part due to the lack of culturable strains and the difficulty of cultivating them. This study demonstrated, for the first time, that all four tested oligotrophic SAR92 clade *Gammaproteobacteria* catabolized DMSP through the DddD-mediated cleavage pathway generating DMS, and that three of these cultivable strains used both the cleavage and demethylation pathways. Considering the wide distribution and relatively high abundance of SAR92 clade bacteria and their *dddD* and *dmdA* genes and transcripts in the global oceans, particularly in polar regions, these bacteria should be considered as an important group of DMSP-degrading bacteria, which are likely significant sources of the climate-active gases DMS and MeSH, and that have been overlooked.

The relative abundance estimates in the *Tara* and polar ocean metagenomes in this study showed a cosmopolitan distribution of the SAR92 clade bacteria in the global oceans. Such a wide distribution of this clade in oceans coincides with the fact that the 16S rRNA gene clones or culturable strains of this clade have been recovered from coastal and pelagic seawater of different sites around the world (1, 2, 47–49). However, unlike the SAR11 clade, the relative abundance of the SAR92 clade is very uneven amongst various sampling sites, indicating that their growth is likely greatly affected by environmental conditions. A higher abundance of this clade is often observed in polar and coastal sea sites usually containing more DMSP than other sea areas (12), implying that it plays a more important role in DMSP degradation in such systems.

The presence of DMSP catabolic gene clusters, whose transcription is upregulated by DMSP substrate, likely allows the SAR92 clade bacteria to respond better to increased DMSP levels compared to many *Roseobacter* and SAR11 clade *Alphaproteobacteria*, which with some exceptions (25), cannot use DMSP as a carbon source (17, 32). Indeed, many *Roseobacter* and SAR11 clade bacteria possess DmdA and DMSP lyase genes (e.g., *dddP*, *dddQ,* and *dddK*), whose transcription is not highly responsive to DMSP substrate, and these bacteria may only use DMSP as a sulfur source or for signaling purposes at low concentrations (32, 50). The ability of SAR92 clade bacteria to utilize DMSP at high concentrations may confer a competitive advantage during algal blooms that can cause extremely high seawater DMSP levels and may explain why SAR92 clade bacteria are abundant in algal bloom samples.

Given that three out of four test strains were found to contain both DMSP demethylation and cleavage pathways and that *dddD* and *dmdA* genes were simultaneously found in many MAGs belonging to the SAR92 clade, both these pathways are likely prevalent in the SAR92 clade bacteria. Co-existence of the DMSP demethylation and cleavage pathways in SAR92 clade bacteria may be advantageous for them to adapt to environments with dynamic DMSP concentrations (34). The predominance of the DMSP cleavage pathway in the three tested strains was observed under our experimental conditions, supporting the hypothesis that marine bacteria favor DMSP cleavage over DMSP demethylation with high DMSP concentrations (16, 51). The transcription of the DMSP demethylation and cleavage genes in these strains could be simultaneously induced by the DMSP substrate, indicating that the SAR92 bacteria regulate both pathways by adjusting the relative expression of the two pathways (52). It has been reported that bacteria probably primarily use DMSP as a carbon source in Arctic seawater, causing high DMS yields from DMSP (53). Given the high abundance of the SAR92 clade bacteria in polar seas, they probably also show the predominance of the DMSP cleavage pathway under *in situ* conditions of Arctic (polar) seas. Moreover, whether and how environmental factors (e.g., light, temperature, and salinity) (54, 55) affect the regulation of the two competing pathways in the SAR92 clade bacteria, finally influencing the fate of DMSP and the release of climate-active gases, remains unclear and merits further investigation.

## MATERIALS AND METHODS

### Water sample collection

Surface seawater samples were collected from two sites (36.41° N, 120.92° E and 37.16° N, 122.59° E) on the coasts (Yellow Sea) of Qingdao and Rongcheng cities, Shandong Province, China. Seawater samples were stored in sterile tubes at 4°C for less than 1 day before processing in the lab.

### Isolation and identification of SAR92 clade strains

A high-throughput culturing technique was used to isolate bacteria from the seawater samples (47). The cell density of the samples was determined by a flow cytometer (Guava EasyCyte HT, Millipore). The samples were diluted to 1.0–5.0 cells/mL in a screening medium (Table S6) and further distributed as 1 mL aliquots into 48-well cell culture plates and incubated in the dark at 15°C for 3 weeks. The cultures in different wells with cell densities above 10^5^ cells/mL checked by flow cytometry were selected and identified based on their 16S rRNA genes, which were amplified using the primers 27F and 1492R. The cultures belonging to the SAR92 clade were chosen to be further purified by several rounds of dilution-to-extinction culturing.

### Genome sequencing and gene annotation

Cells of each isolated SAR92 clade strain grown in 1 L screening medium for 5 days were collected by a 0.2 µm polycarbonate membrane (Isopore, Merck Millipore, USA). Genomic DNAs of these strains were extracted using a DNA extraction kit (Qiagen, Hilden, Germany) and sequenced through an Illumina Hiseq platform. Genome assembly was performed using ABySS v2.0.2 with multiple Kmer parameters. The genome sequence of HTCC2207 was downloaded from the NCBI (the National Center for Biotechnology Information) database. All the genomes were annotated using the RAST server2 (56). The BLASTp program was used to predict the putative enzymes with the thresholds of coverage of 80%, similarity of 30%, and *E*-value e^−10^.

### Phylogenetic analysis of the SAR92 strains

The 16S rRNA gene sequences of cultured and uncultured clones and closely related species as well as genomes of SAR92 strains were obtained from NCBI database. The neighbor-joining phylogenetic trees based on the 16S rRNA gene sequences were reconstructed using MEGA X. Bootstrap analyses were performed based on 1,000 replicates to estimate the confidence levels of the branches in the phylogenetic trees generated.

### Analysis of the ability of SAR92 clade strains to utilize DMSP and related compounds

SAR92 clade strains were grown in artificial seawater medium AMS1 (57), which contained 9 mM KCl, 10 mM CaCl_2_·2H_2_O, 27 mM MgCl_2_·6H_2_O, 2.8 mM MgSO_4_·7H_2_O, 481 mM NaCl, 6 mM NaHCO_3_, macronutrients, trace metals, and a vitamin mix (Table S6). To test the ability of SAR92 clade strains to utilize DMSP as a sole carbon source, cells were grown in AMS1 amended with 10 µg/mL D-glucose before inoculation. Then, cells of each strain were collected and inoculated in AMS1 amended with 50 µM DMSP hydrochloride (TCI, Japan). The initial cell density in the medium was approximately 10^4^ cells/mL. The positive control was amended with 50 µM pyruvate instead of DMSP, while no carbon sources were added to the negative control culture. To test the ability of SAR92 clade strains to utilize DMSP-related compounds, cells were inoculated in AMS1 amended with 100 µM of each compound (3HP, DMS) as a sole carbon source. The same medium without the addition of the carbon source was used as the negative control. To test the ability of SAR92 clade strains to utilize DMSP as a sole sulfur source, strains were grown in a medium without the addition of sulfur (9 mM KCl, 10 mM CaCl_2_·2H_2_O, 30 mM MgCl_2_·6H_2_O, 418 mM NaCl, 6 mM NaHCO_3_, 0.8 mM NH_4_Cl, 50 µM NaH_2_PO_4_, trace metals, and vitamin mix), then supplied with 50 µM DMSP or methionine (positive control) before inoculation. The same medium with no sulfur source added was used as the negative control. Each treatment included three replicates and cultures were grown in an incubator at 16°C under dark conditions. Cell densities were monitored with a Guava EasyCyte HT flow cytometer using Guava InCyte v3.1 software. The instrument and software were set up as follows: channel 01, bright field; channel 02, fluorescence channel (Green-B fluorescence: excitation wavelength, 488 nm); and flow rate, low speed. For the analysis, cells were stained with SYBR Green I (Solarbio) (final dilution 1: 2,000) for 40 min at room temperature in the dark.

### Measurement of DMS and MeSH

SAR92 clade strains were grown in modified AMS1 amended with 10 µg/mL D-glucose. Cells were collected by centrifugation and resuspended in modified AMS1 with the final concentration of >10^8^ cells/mL. Then, the cell suspension was supplied with 1 mM DMSP and distributed into gas-tight sealing bottles (1 mL per vial), which were incubated at 16°C for 5 days. The mixture without DMSP and the mixture without bacteria were set as negative controls. DMS and MeSH produced in the mixture were measured using gas chromatography (GC-2030, Shimadzu, Japan) equipped with a flame photometric detector and a fused silica capillary column (30 m × 0.53 mm × 1 µm) as described by Zhang et al. (58). In brief, the sample gas was injected into GC using a purge-and-trap device. Nitrogen was used as the carrier gas. The column temperature was 70°C and the detector temperature was 250°C. A two-sided Student’s *t* test was used to analyze whether the production of DMS and MeSH was statistically significantly different.

### Real-time qPCR analysis

Cells were cultured in modified AMS1 amended with 10 µg/mL D-glucose to the cell density of 1.0 × 10^7^ mL^−1^. Then, cells were induced by 1 mM DMSP for 1, 3, and 6 h, and the control group without DMSP was also set up. Total RNA was extracted using an RNeasy Mini Kit (Qiagen Biotech, Germany) and was subsequently reverse transcribed to cDNA using a PrimeScript RT reagent Kit (TransGen, China). The qPCR was performed on the LightCycler 480 System (Roche, Switzerland) using a SYBR Premix Ex Taq (Takara, Japan). Relative expression levels of target genes were calculated using the LightCycler 480 software with the “advanced relative quantification” method. The *recA* was used as an internal reference gene. The primers used are shown in Table S7.

### Gene cloning, protein expression, and purification

Genes of interest were amplified and cloned from the SAR92 clade strain genomes by PCR using *FastPfu* DNA polymerase (TransGen Biotech, China). The amplified genes were digested and cloned into the *Nde*I/*Xho*I restriction sites of the pET-22b vector (Novagen, Germany) to incorporate a C-terminal His tag. Cloned genes in pET-22b were overexpressed in *Escherichia coli* BL21(DE3). The recombinant *E. coli* BL21(DE3) cells were cultured in the Luria-Bertani (LB) medium supplemented with 0.1 mg/mL ampicillin at 37°C to an OD_600_ of 0.8–1.0 and then induced at 18°C for 16 h with 0.5 mM isopropyl-β-D-thiogalactopyranoside. After induction, cells were collected by centrifugation, resuspended in the lysis buffer (50 mM Tris-HCl, 100 mM NaCl, 0.5% glycerol, pH 8.0), and fractured by pressure crusher. Recombinant proteins were purified with Ni^2+^-NTA resin (Qiagen, Germany) and eluted with elution buffer (50 mM Tris-HCl, 100 mM NaCl, 350 mM imidazole, 0.5% glycerol, pH 8.0), followed by desalination on PD-10 Desalting Columns (GE Healthcare, USA) equilibrated with 10 mM of Tris-HCl, pH 8.0 and 100 mM of NaCl.

### Enzyme assays

The enzymatic activity of DddD toward DMSP was determined according to the method described by Alcolombri et al. (31). In brief, the DddD protein (5 mg/mL) and cofactor acetyl-CoA (1 mM) were added in the reaction mixture of 1 mM DMSP and 100 mM Tris-HCl (pH 8.0). The reaction was performed at 30°C for 1 h and terminated by adding 10% (vol/vol) HCl. The control groups had the same reaction system except that the DddD protein was not added. The DMS production was detected by GC as described above.

### Complementation of the *Ruegeria pomeroyi* DSS-3 Δ*dmdA* mutant

For complementation of the *R. pomeroyi* DSS-3 Δ*dmdA* mutant, the *dmdA* homologs with their native promoters from strains H921, H231, H455, and HTCC2207 were amplified, digested with *EcoR* I and *Xho* I, and cloned into the vector pHG101 (59) to generate pHG101-*dmdA*_H921_, pHG101-*dmdA*_H231_, pHG101-*dmdA*_H455_, and pHG101-*dmdA*_HTCC2207_, respectively. These plasmids were then transformed into *E. coli* WM3064 and mobilized into the Δ*dmdA* mutant by conjugation, respectively. After conjugation, the cells were plated on the Marine Agar 2216 plates containing kanamycin (100 µg/mL) to select for the pHG101 plasmid. Colony PCR was used to confirm the presence of the transferred plasmid. The strains, plasmids, and primers used in this study are shown in Tables S8 and S9. The obtained *dmdA*-complemented strains were grown in Marine Broth 2216 medium containing kanamycin and assayed for the MeSH production using GC.

### Bioinformatic analysis

Raw metagenome sequence data were obtained from 132 samples from 60 stations of the *Tara* Ocean project and 60 samples from 28 stations of the Polar Ocean Project (60). The raw data were filtered and assembled using the default pipeline of metaWRAP, and then the clean data were annotated for classification using Kraken2 (NR database, cut-off date: 1 March 2022) (61).

To investigate the distribution of *dddD* and *dmdA* belonging to the SAR92 clade in the gene sets, HMM were constructed using functionally verified DddD and DmdA protein sequences (Table S10) and used to search for homologous sequences in *Tara* or polar ocean databases using HMMER 3.3.1 (*E*-value, 1e^-30^). The obtained homologous sequences were blasted using BLASTp against the local non-redundant protein sequences (NR database, cut-off date: 1 March 2022) databases. The taxon of the best hit to a query sequence was used to determine the species information of the query sequence. Sequences belonging to the SAR92 clade were retained for further analysis. The gene (or transcript) abundance estimates were expressed by the gene’s (or transcript’s) read coverage divided by the sum of the total gene (or transcript) coverages for the sample (“percentage of total coverage”) (62). Subsequent data processing and visualization were performed based on R software.

## Data Availability

The main data supporting the findings of this study are available in the article and in its supplemental information. All data and materials supporting the findings of this study are available from the corresponding authors upon reasonable request. The whole-genome sequences of strains H455, H231, and H921 in this study are publicly available from the NCBI's GenBank with accession numbers CP103416, JAOALC000000000, and JAOALD000000000, respectively.

## SUPPLEMENTAL MATERIAL

Supplemental figures mbio.01467-23-s0001.pdf Figures S1 to S13.

Supplemental tables mbio.01467-23-s0002.xlsx Tables S1 to S10.

ASM does not own the copyrights to Supplemental Material that may be linked to, or accessed through, an article. The authors have granted ASM a non-exclusive, world-wide license to publish the Supplemental Material files. Please contact the corresponding author directly for reuse.
