# Supplementary material for: SAR92 clade bacteria are potentially important DMSP degraders and sources of climate-active gases in marine environments
Source: mBio. 2023 Nov 10;14(6):e01467-23. doi: 10.1128/mbio.01467-23 (PMC10746254; doi:10.1128/mbio.01467-23)
Supplement: Supplemental figures — Figures S1 to S13. [file mbio.01467-23-s0001.pdf]

SAR92 clade bacteria are potentially important DMSP degraders and sources of climate-active gases in marine environments

Xiao-Yan He[1,2,3,7], Ning-Hua Liu[1,3,7], Ji-Qing Liu[1], Ming Peng[1], Zhao-Jie Teng[1], Tie-Ji Gu[1], Xiu-Lan Chen[1,3], Yin Chen[2,4], Peng Wang[2,3], Chun-Yang Li[2,3], Jonathan D. Todd[5], Yu-Zhong Zhang[2,3,6]*, Xi-Ying Zhang[1,3]*

[1]State Key Laboratory of Microbial Technology, Shandong University, Qingdao, China

[2]MOE Key Laboratory of Evolution and Marine Biodiversity, Frontiers Science Center for Deep Ocean Multispheres and Earth System & College of Marine Life Sciences, Ocean University of China, Qingdao, China

[3]Laboratory for Marine Biology and Biotechnology, Laoshan Laboratory, Qingdao, China

[4]School of Life Sciences, University of Warwick, Coventry, UK

[5]School of Biological Sciences, University of East Anglia, Norwich Research Park, Norwich, UK

[6]State Key Laboratory of Microbial Technology & Marine Biotechnology Research Center, Shandong University, Qingdao, China.

[7]These authors contributed equally: Xiao-Yan He, Ning-Hua Liu

*Corresponding authors: Xi-Ying Zhang, zhangxiying@sdu.edu.cn; Yu-Zhong Zhang, zhangyz@sdu.edu.cn

Running title: DMSP catabolism in SAR92 clade bacteria

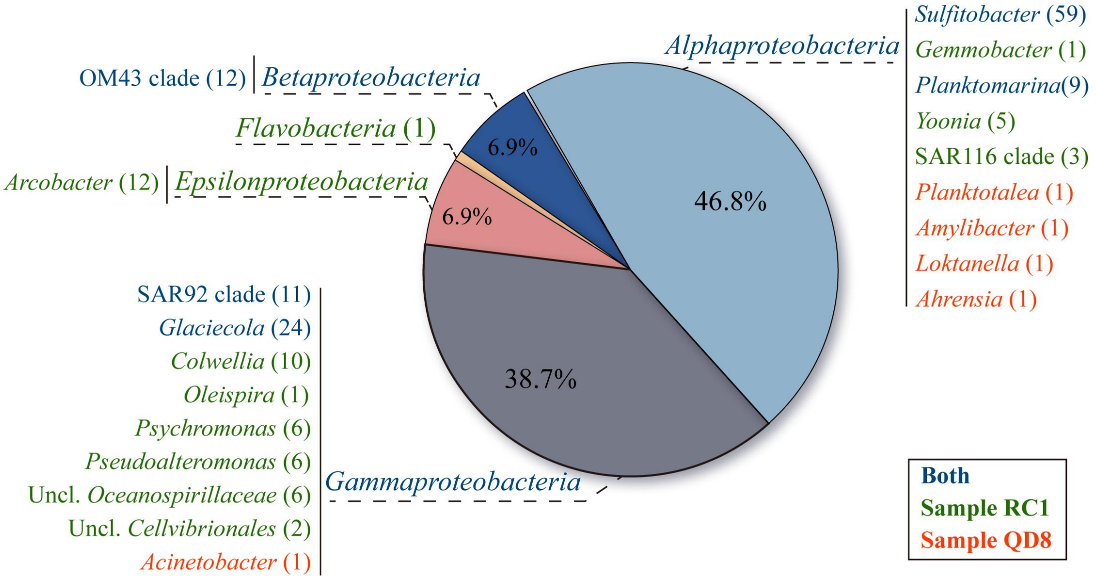

FIGURE S1. Taxonomic compositions of the strains isolated through high-throughput cultivation. Abundance of the culturable bacteria isolated from two seawater samples (RC1 and QD8) collected from the coast of Shandong province, China. Colors indicate the source of isolated strains belonging to the different bacterial genera: the sample QD8 only (red); the sample RC1 only (green), both of the samples (blue).

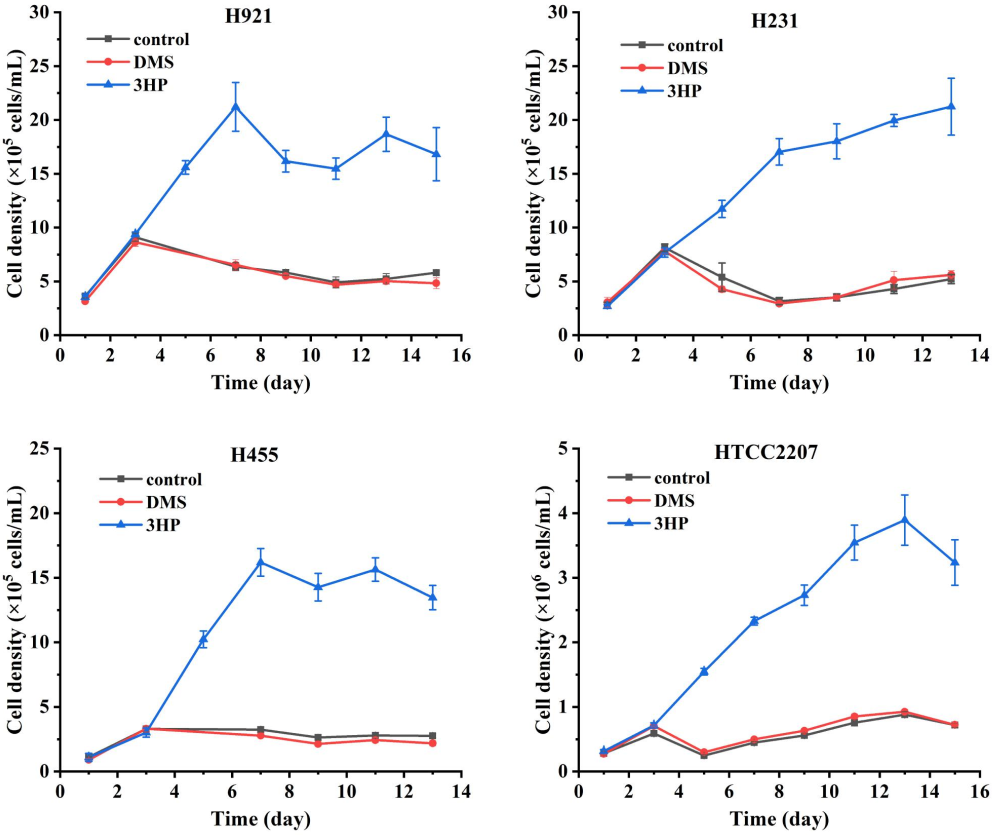

FIGURE S2. The growth curves of SAR92 strains on DMSP metabolic intermediates (3HP, DMS). Strains H921, H231, H455 and HTCC2207 were grown at 16°C in the AMS1 medium amended with 100 μM 3HP or DMS as the sole carbon source, culture without any carbon source was used as the negative control.

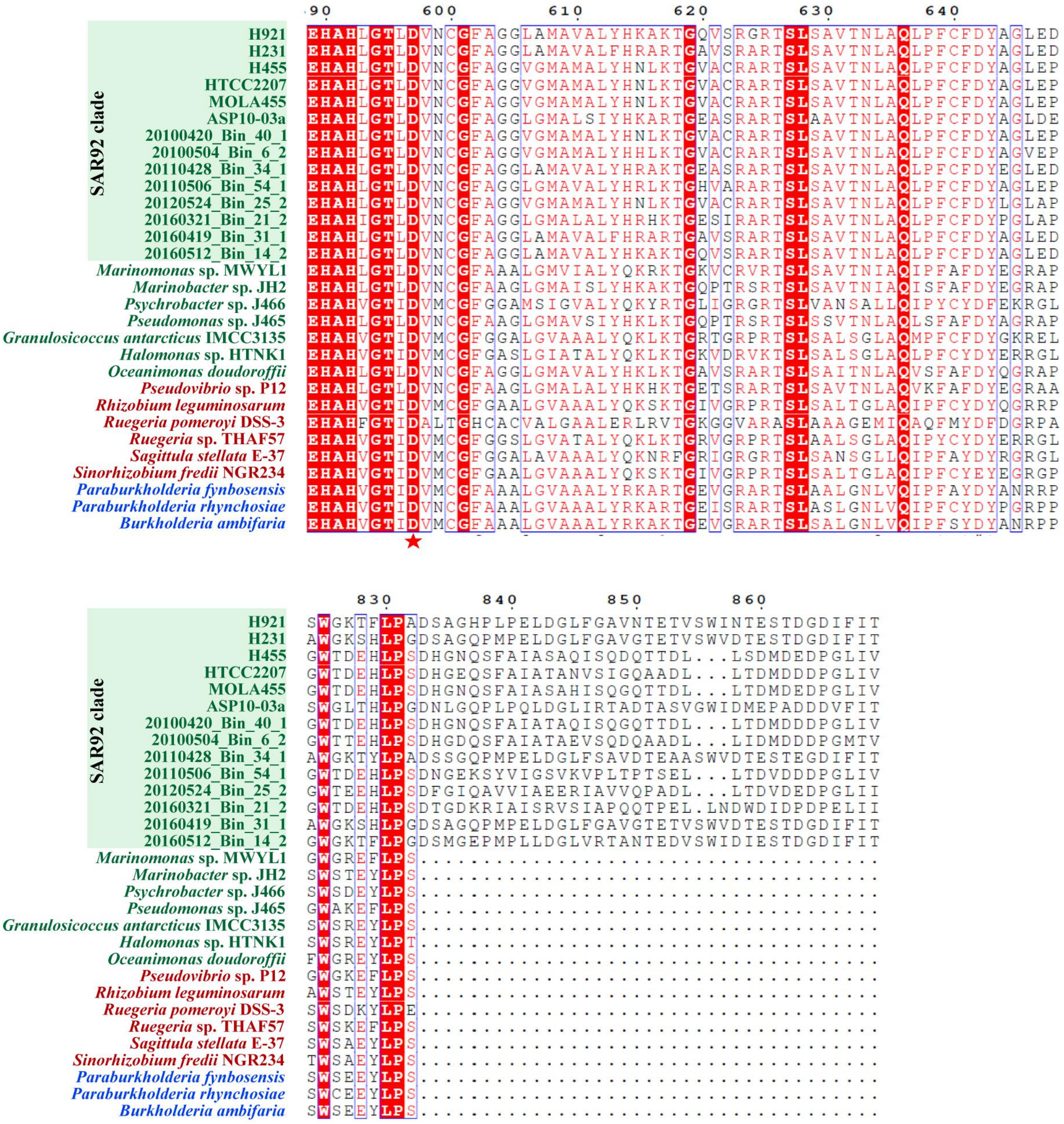

FIGURE S3. Amino acid sequence alignment of the C-terminals of DddD DMSP lyases of strains or MAGs belonging to the SAR92 clade and other groups. DddDs from *Gammaproteobacteria* (green), *Alphaproteobacteria* (red), and *Betaproteobacteria* (blue) are shown in different colors. Identical residues are marked with red filled boxes. The catalytic residue is marked with a red star.

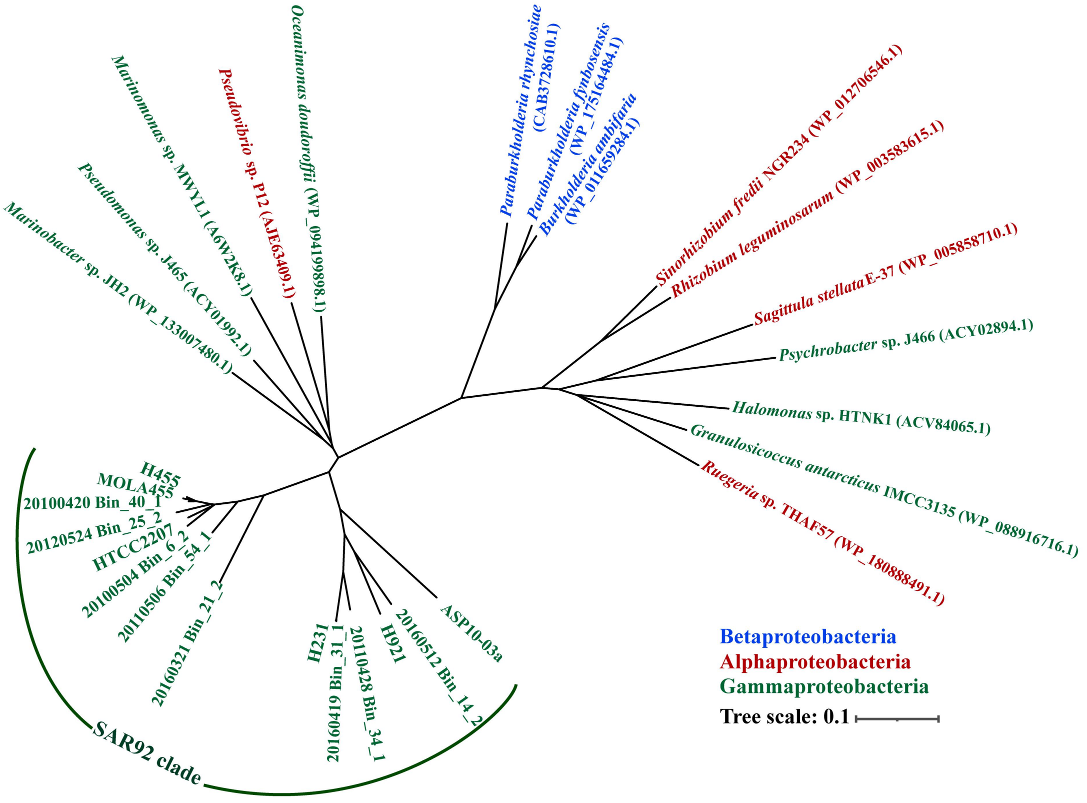

FIGURE S4. Phylogenetic tree based on amino acid sequences showing positions of the DddD DMSP lyases from strains or MAGs in the SAR92 clade separate from those of strains in other groups. DddDs from *Gammaproteobacteria* (green), *Alphaproteobacteria* (red), and *Betaproteobacteria* (blue) are shown in different colors.

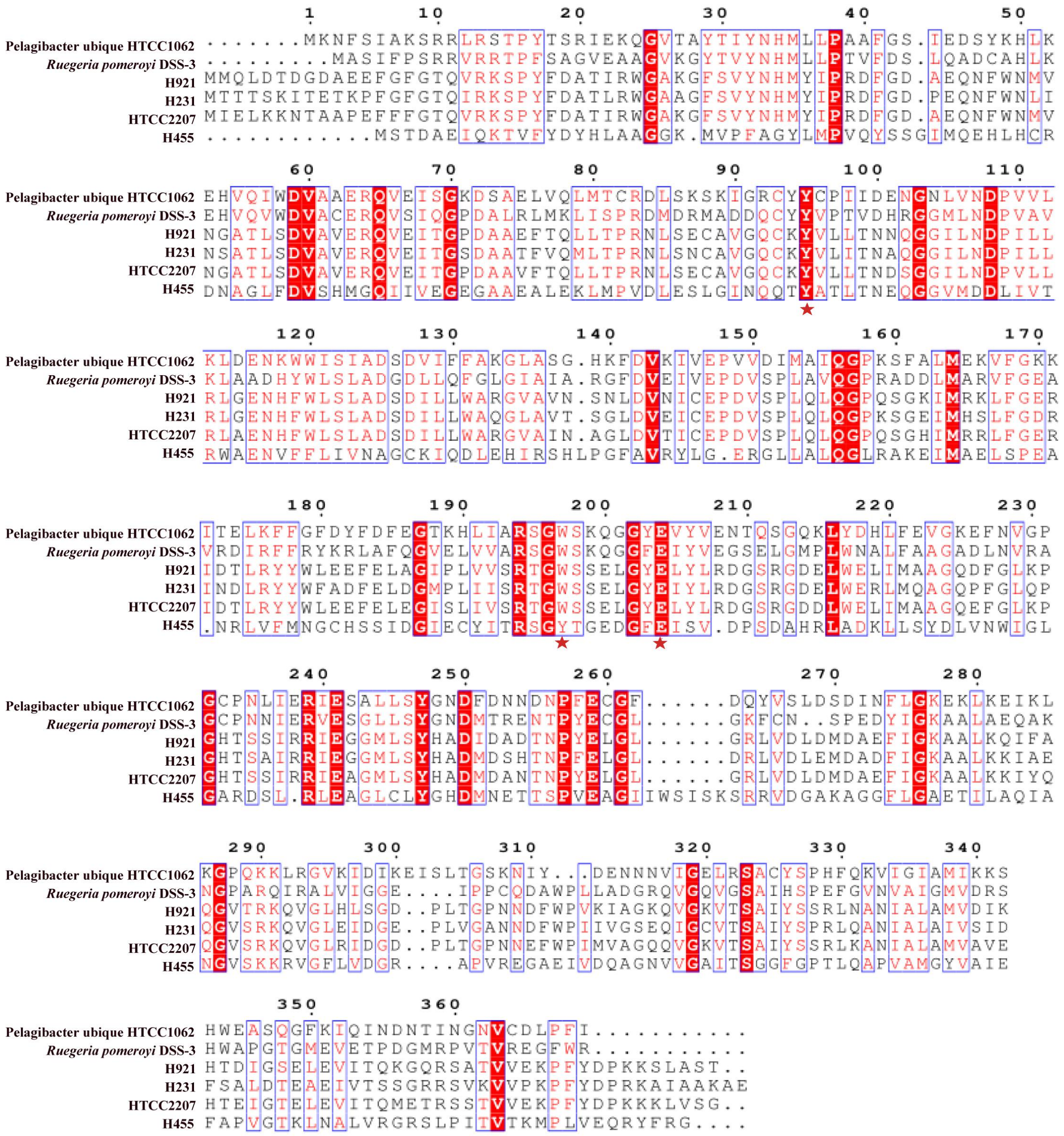

FIGURE S5. Amino acid sequence alignment of the DmdA homologs of SAR92 clade strains and two functional DmdAs.

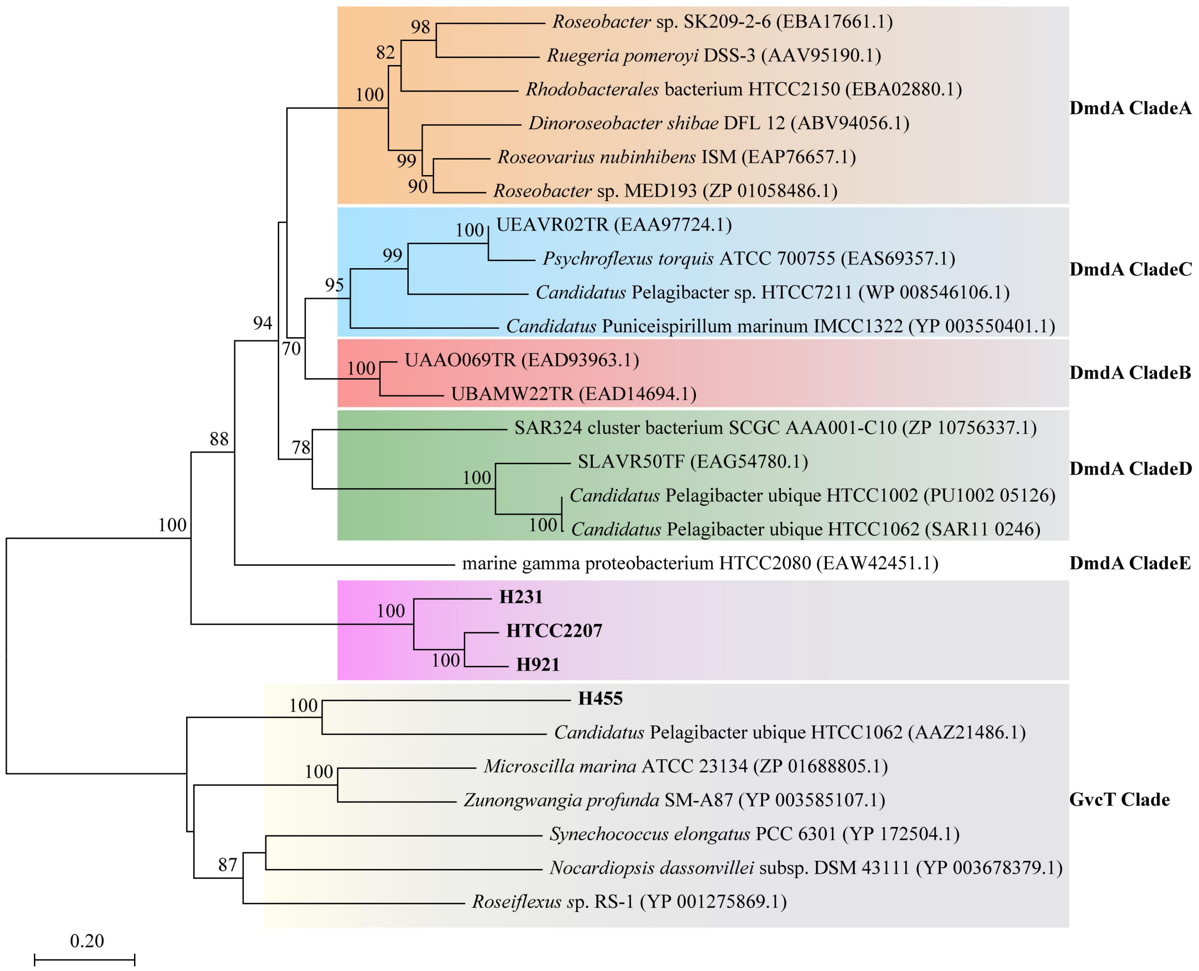

FIGURE S6. Phylogenetic tree based on amino acid sequences showing positions of the DmdA homologs from SAR92 clade strains separate from those of strains in other groups.

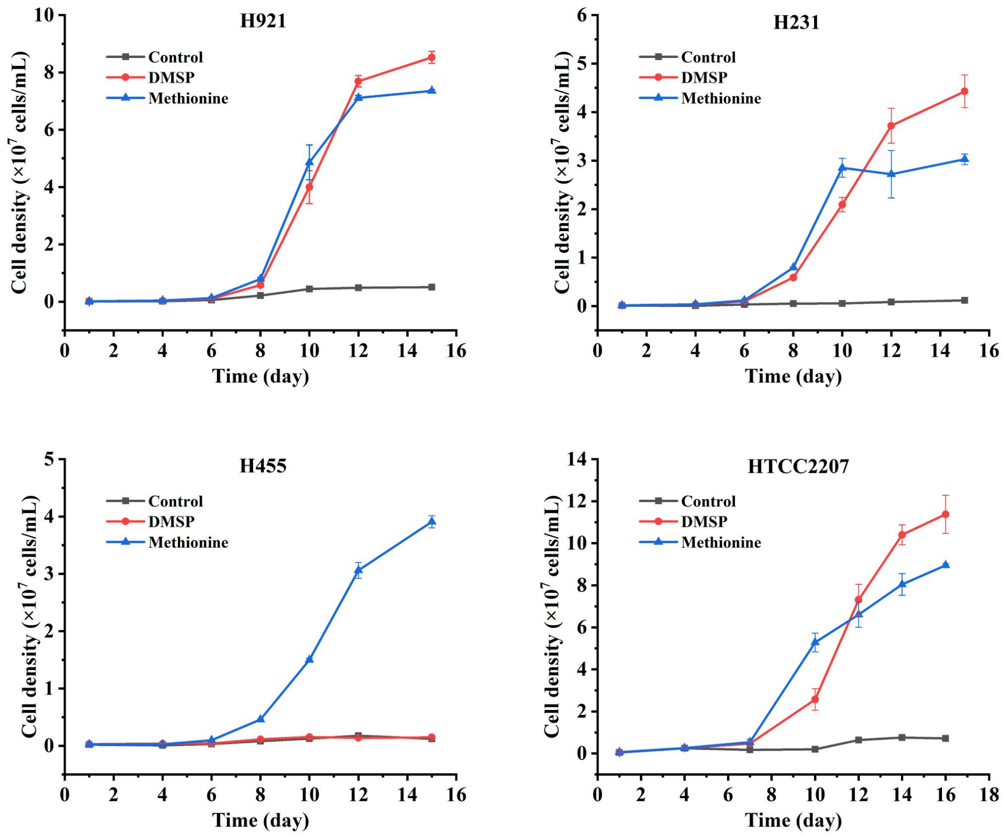

FIGURE S7. The growth curves of SAR92 strains on DMSP as sulfur source. Strains H921, H231, H455 and HTCC2207 were grown at 16°C in the dark with 50 μM DMSP as the sole sulfur source, culture without any sulfur source was used as the negative control.

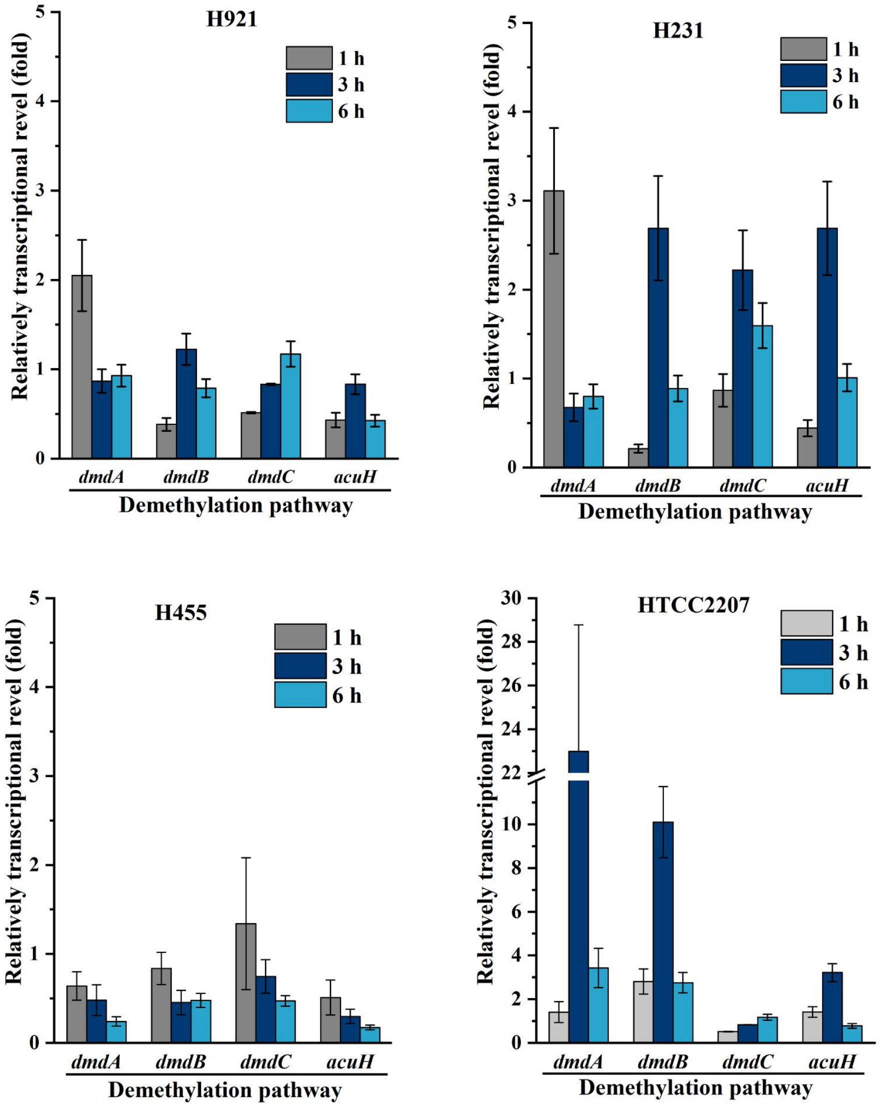

FIGURE S8. Transcriptions of the genes involved in DMSP demethylation pathway in the SAR92 clade strains. RT-qPCR assay of the transcriptions of the genes involved in the demethylation pathways from strains H921, H231, H455 and HTCC2207 in response to DMSP in the AMS1 medium. The bacteria cultured in the same medium without DMSP were used as the control. The *recA* gene was used as an internal reference. The error bars represent standard deviation of triplicate experiments.

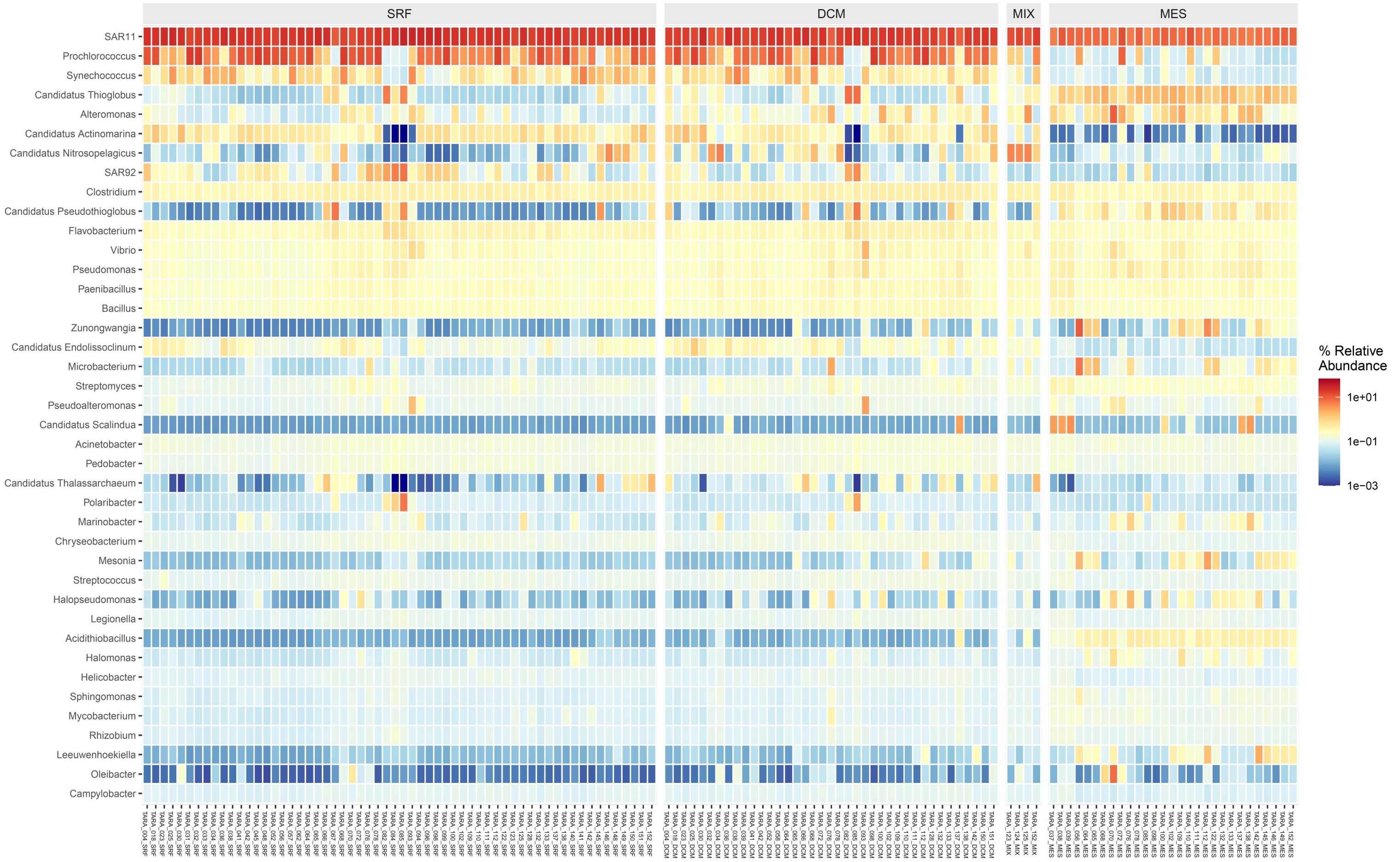

FIGURE S9. The relative abundance of different genera or clades in *Tara* ocean metagenomes.
Metagenomic samples are separated into four groups according to the environmental features of the sampling sites: SRF (surface water layer), DCM (deep chlorophyll maximum layer), MES (mesopelagic zone) and MIX (marine epipelagic mixed layer)

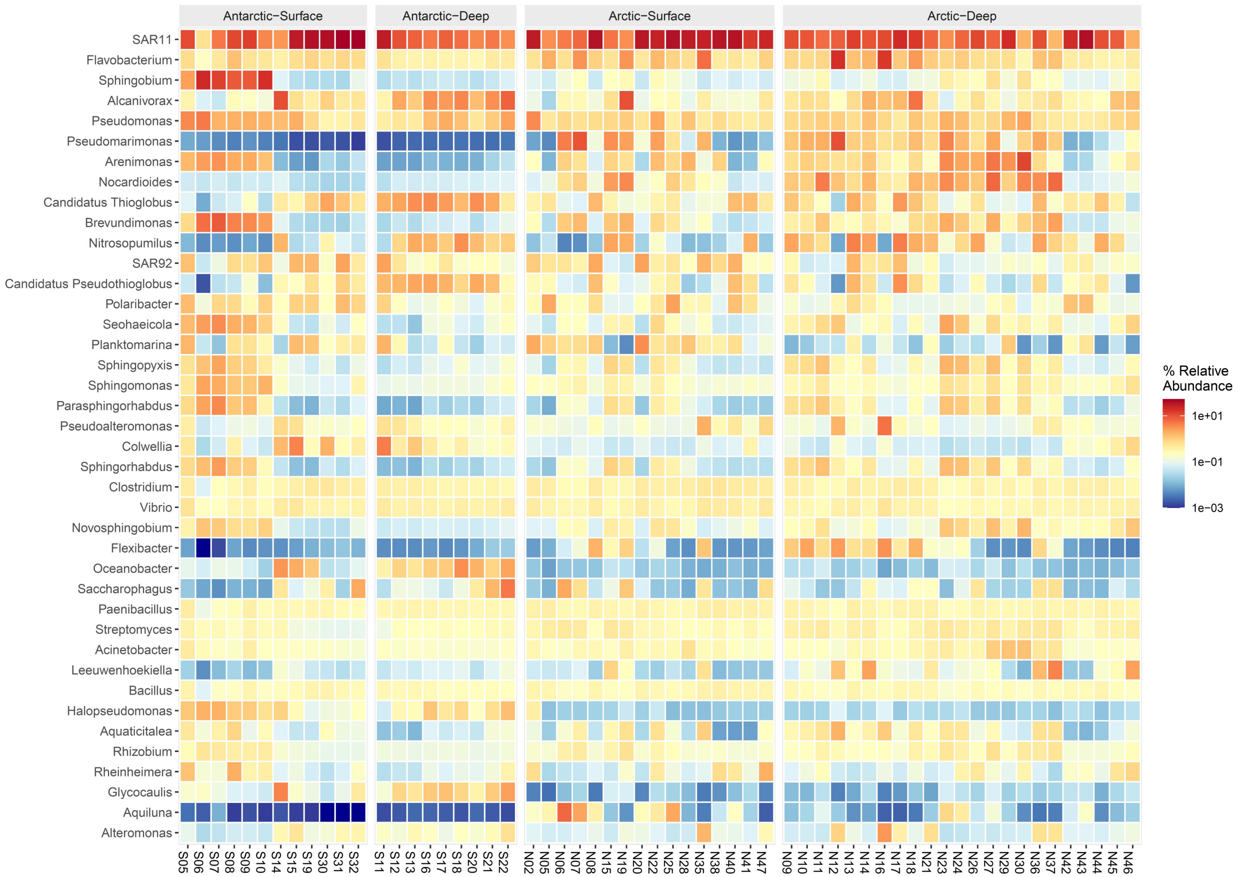

FIGURE S10. The relative abundance of different genera or clades in polar ocean metagenomes

Metagenomic samples are separated into four groups according to the environmental features of the sampling sites: Arctic-Surface, Arctic deep, Antarctic-Surface and Antarctic-deep

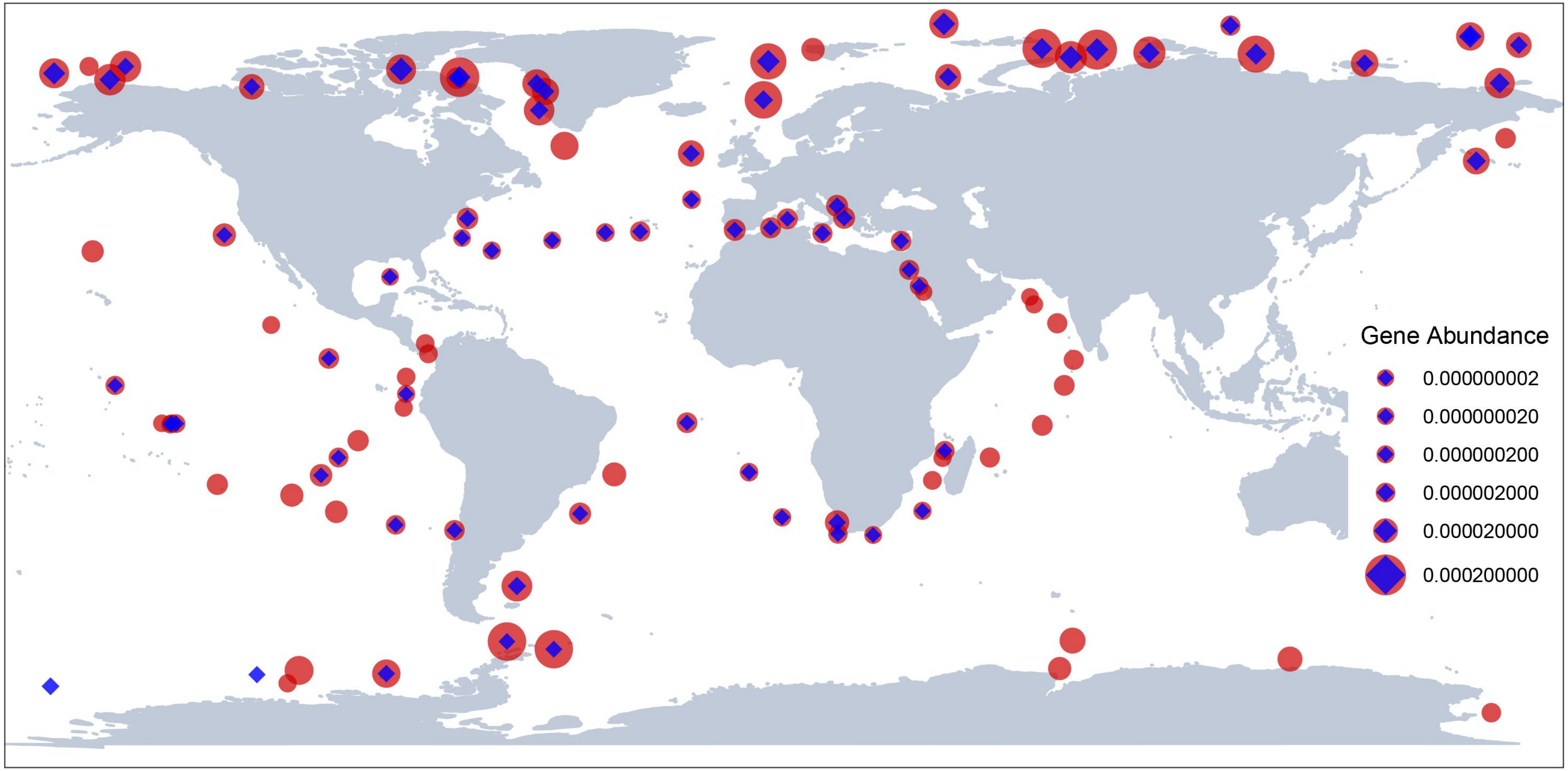

FIGURE S11. Distribution of the *dmdA* (●) and *dddD* (◆) genes belonging to the SAR92 clade in the metagenomes in *Tara* and polar ocean databases.

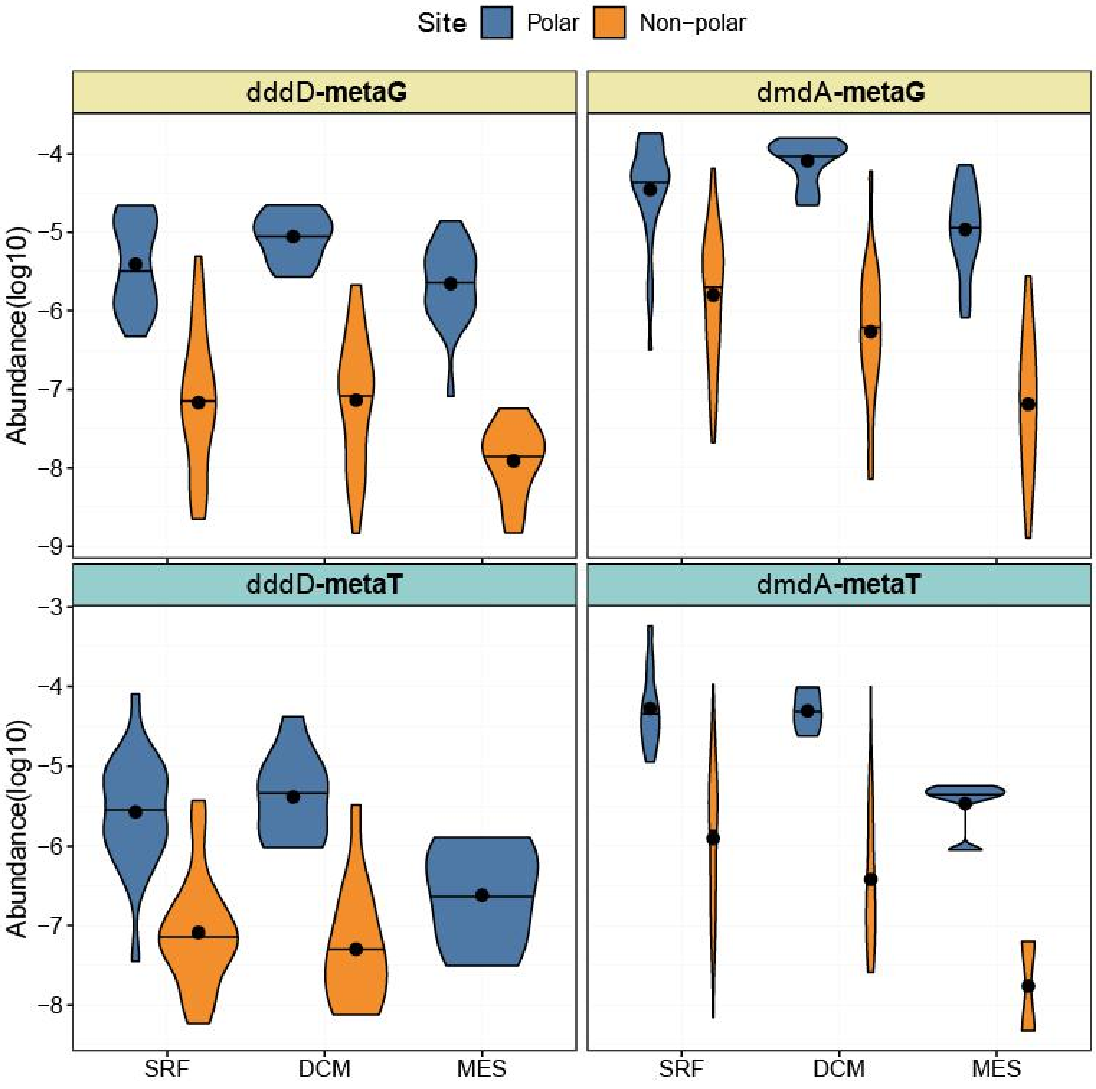

FIGURE S12. Abundance of the *dmdA* and *dddD* genes belonging to the SAR92 clade in the metagenomes and the metatranscriptomes of different layer seawater samples in *Tara* and polar ocean databases. MetaG, metagenomes; MetaT, metatranscriptomes; SRF, surface seawater layer; DCM, deep chlorophyll maximum layer; MES, mesopelagic layer.

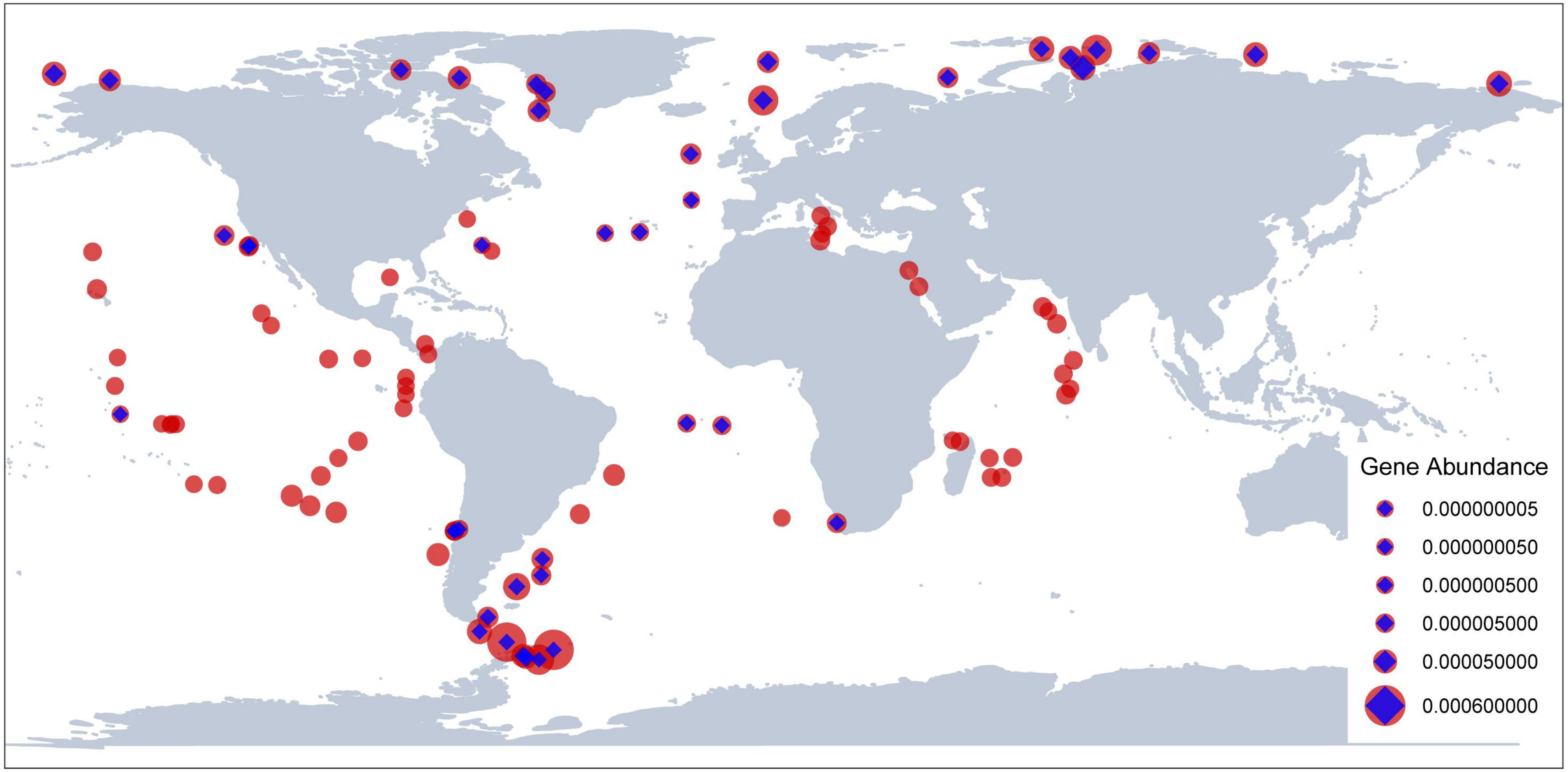

FIGURE S13. Distribution of the *dmdA* (●)and *dddD* (◆)transcripts belonging to the SAR92 clade in the metatranscriptomes in *Tara* database
